# Electrically controllable laser frequency combs in graphene-fibre microresonators

**DOI:** 10.1038/s41377-020-00419-z

**Published:** 2020-11-09

**Authors:** Chenye Qin, Kunpeng Jia, Qianyuan Li, Teng Tan, Xiaohan Wang, Yanhong Guo, Shu-Wei Huang, Yuan Liu, Shining Zhu, Zhenda Xie, Yunjiang Rao, Baicheng Yao

**Affiliations:** 1 Key Laboratory of Optical Fibre Sensing and Communications (Education Ministry of China), University of Electronic Science and Technology of China, Chengdu, China; 2 National Laboratory of Solid State Microstructures and, School of Electronic Science and Engineering, School of Physics and College of Engineering and Applied Sciences, Nanjing University, Nanjing, China; 3 Department of Electrical, Computer, and Energy Engineering, University of Colorado Boulder, Boulder, CO 80309 USA; 4 Key Laboratory for Micro-Nano Optoelectronic Devices (Education Ministry of China), School of Physics and Electronics, Hunan University, Changsha, China; 5 Research Centre for Optical Fibre Sensing, Zhejiang Laboratory, Hangzhou, China

**Keywords:** Frequency combs, Mode-locked lasers, Optoelectronic devices and components, Optical properties and devices

Dear Editor,

Laser frequency combs emitting ultrafast pulses of light, at equidistantly discrete frequencies, are cornerstones of modern information networks. In recent years, the generation of soliton combs in microcavities with ultrahigh-quality factors has established microcombs as out-of-laboratory tools. However, the material and geometry of a laser cavity, which determine comb formation, are difficult to electrically tune. Such dynamic control can further enrich the diversity of comb outputs and help to actively stabilize them. Here we demonstrate electrically controllable laser frequency combs in a heterogeneous graphene-fibre microcavity. By altering the Fermi level of atomically thick graphene, we simultaneously demonstrate the tunable absorption, controllable *Q*-factor, and fast optoelectronic feedback stabilization. Thus, we can use this device to produce mode-locked laser combs with tunable repetition rates, controllable wavelengths, and self-stabilized phase noise down to −120 dBc Hz^−1^ at 10 kHz. The span of the combs can be further broadened to more than half an octave through convenient supercontinuum amplification. Combining phase-locking techniques and single-atomic-layer optoelectronics, this study provides knowledge for achieving a high-repetition rate optical frequency comb on fibre.

The development of laser frequency combs has revolutionized optical communication, photonic sensing, precision spectroscopy, and astronomical observation^1,2^. Stable frequency combs can be achieved via mode locking in rare-earth doped fibre lasers^3,4^, generating Kerr solitons in parametric oscillators^5–8^, or opto-electrically modulating lithium niobate microresonators with strong second-order nonlinearity^9,10^. For many out-of-lab applications, people desire a compact comb device with multiple enhancements, such as an all-in-fibre integration^11^, low-driven power but high efficiency^12,13^, full stabilization^14,15^, and diverse comb outputs with fast and convenient tunability^16^. The rise of graphene has spurred unprecedented advances ranging from material physics, to mechanics, optoelectronics, and biomedicines^17–19^. In optics, the symmetrically gapless dispersion of the quasiparticle Dirac fermions of graphene renders its optical permittivity to be defined only by the fine-structure constant^20^. This unique feature leads to a remarkable tunability in nonlinear absorption and phase shift^21,22^, thus enabling diverse optoelectronic modulators^23–25^, passively mode-locked lasers^26–29^, and photonic sensors^30–32^. Via tuning the carrier density electrically, graphene devices also enable distinct lasing tunability either in-fibre cavities or microresonators^16,33^. Unlike conventional graphene used in passively mode-locked lasers, we explore a new geometry in which we integrate a graphene *p*–*n* junction^34^ in an erbium/ytterbium fibre-based Fabry–Perot microcavity via the van der Waals assembly technique^35^. The all-in-fibre configuration enables the generation of high-repetition laser combs with unique electrical tunability and feedback stabilization in situ.

Figure 1a, b show the concept and characterization of our heterogeneous graphene Fabry–Perot resonator (GFPR). This GFPR consists of a 1 cm-long erbium/ytterbium co-doped single-mode fibre, which can be directly driven by a standard 980 nm laser. The end facets of the resonator are coated with ten-layer pairs of Ta_2_O_5_ and SiO_2_ using the ion-assisted deposition method. These dielectric Bragg reflectors have a thickness of 4.5 μm, with a high reflectivity of over 98%, enabling the maximum intrinsic *Q* > 2 × 10^6^, whereas the erbium/ytterbium-based absorption is ≈2%. The group velocity dispersion of the fibre section is anomalous in the C + L band, which is helpful for dissipative soliton formation. Thus, we do not need to chirp the dielectric Bragg reflectors for an additional chromatic dispersion composition. The monocrystalline single-layer graphene is grown by the chemical vapour deposition (CVD) method and connected by Ti/Au electrodes (5 nm/50 nm), forming a current driven *p*–*n* junction, which can be directly driven by a source–drain voltage (*V*_SD_). The semiconductor channel width of graphene is ~60 μm. Different from the vertically gated graphene field-effect transistors (FETs) in previous studies^16^, this graphene incorporation without a gating layer simplifies the structural implementation for better in-fibre incorporation. It also decreases intracavity optical scattering loss, which is helpful for preserving the *Q*-factor and avoiding the risk of heat damage. Device fabrication and characterizations are further detailed in the ‘Methods’ and Supplementary Section 2. The monolayer graphene coverage provides broadband optical absorption ≈2.3%; thus, we measured that the loaded *Q*-factor of the GFPR was 9.6 × 10^5^ in the lasing band (intrinsic *Q*-factor of 1.5 × 10^6^). Preserving the *Q*-factor at a value higher than one million helps to generate laser combs at a pump power <1 W. In further applications, one can increase the erbium/ytterbium doping rate to obtain a higher laser power, while also promoting the lasing and mode-locking threshold.

**Fig. 1:**
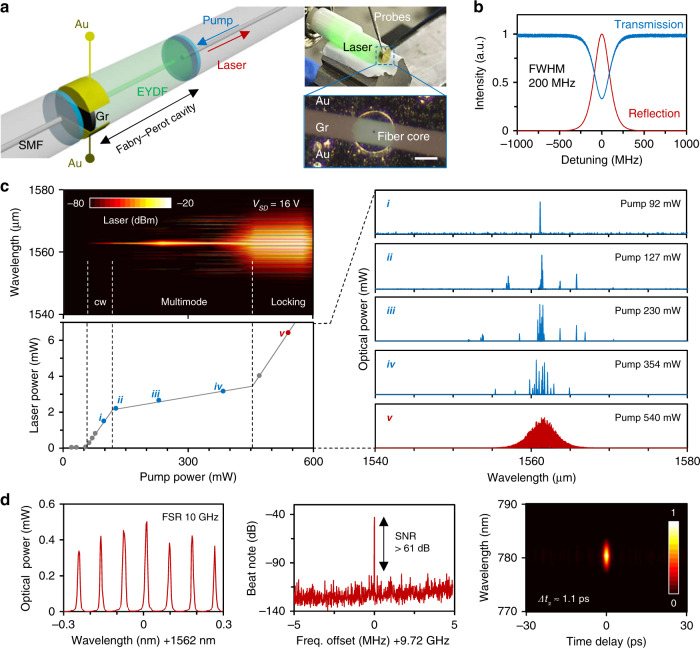
Graphene heterogeneous *F*–*P* fibre laser resonator. **a** Architecture of the implementation. The graphene heterostructure is incorporated on one side of the resonator. Optical micrographs show that we can drive the resonator by using electric probes and green scattering is excited by the gain media (upper panel). Here, the bar is 60 μm (bottom panel). **b** Transmission and reflection of the resonator. **c** Laser evolution (*V*_SD_ = 16 V). By increasing the pump power, the laser operation travels from CW to multimode and then to locking. The laser threshold is ≈60 mW and the mode-locking threshold is ≈450 mW. Curves *i* to *v* plot the measured spectra of the laser states. **d** Characterization of the mode-locked state, from left to right: magnified optical spectrum, the first-order beat note in RF, and the frequency-resolved autocorrelation map

Driven by a 980 nm laser diode (LD) pump, the GFPR generates a laser in the C or L band. Figure 1c shows the laser evolution process when we fix *V*_SD_ = 16 V. By increasing the pump power, the laser finally operates from a continuous wave (cw) state and multimode state to a dissipative soliton mode-locking state^36^. The laser threshold of the GFPR here is ≈60 mW. When the pump power increases to over 100 mW, the laser operates in an unstable multimode state, and laser lines appear at various resonances. Once the pump power is higher than 450 mW, the high intracavity energy density enables mode locking due to the saturable absorption of graphene. In this evolution, the lasing efficiency of the multimode operation is considerably lower than either the cw operation or the mode-locking operation, determined by fierce mode competition. Here we also illustrate the measured spectra of several states, for example (state *i* to state *v*). We discuss the theoretical laser dynamics in Supplementary Section 1. Specifically, we characterize the fundamental mode-locking state (*v*) in Fig. 1d. The optical spectral analysis verifies that the repetition rate of the laser cavity is ≈10 GHz. We also show the first-order beat note of the pulsed laser and the signal-to-noise ratio (SNR) is higher than 61 dB. Moreover, by using the autocorrelation technique, we illustrate the frame-by-frame frequency-resolved second-harmonic autocorrelation map. Limited by the small optical gain and the relatively large third-order dispersion in the fibre, the autocorrelation profile is measured to be 1.1 ps, suggesting that the pulse width is ≈710 fs in the sech^2^ shape. This value matches well to the spectral bandwidth (≈4.5 THz), considering the sech^2^-based time–bandwidth product of 0.315.

Figure 2a schematically shows the electrical tuning of the in-fibre graphene. Initially, when deposited on silica fibre, graphene is naturally *p*-doped (holes are dominant). Once driven by current, external electrons are injected into the graphene sheet. As a result, the Fermi level of the intracavity graphene is tuned from *p*-doping to *n*-doping. Due to Pauli blocking^21,26,27^, graphene shows better optical transparency when the Fermi level is far from the Dirac point. Figure 2b plots the measured voltage-current response and the graphene resistance. The Fermi level originally equals 0.25 eV. When increasing the *V*_SD_, the nonlinear correlation of the source–drain current (*I*_SD_) vs. the *V*_SD_ indicates that the external *V*_SD_ modifies the resistance of the heterostructure from 220 to 3350 Ω. Such tuning enables graphene quasi-Fermi level changing, based on the Drude model *μ* = (*Nρ*)^−1^ and the quasi-Fermi energy equation *|E*_F_ | = *ħ|v*_F_|(*πN*)^−1/2^ ^37^. Here, *μ* is the mobility, *ρ* is the resistance, *N* is the carrier density, *ħ* is Planck’s constant, and *v*_F_ is the Fermi velocity. When the *V*_SD_ approaches 12 V, the Fermi level is close to the Dirac point and further increasing the *V*_SD_ induces *n*-doping. By tuning the *V*_SD_ from 12 to 50 V, we can control the |*E*_F_| from 0 to 0.45 eV. In addition, the graphene *p*–*n* junction is able to control the local temperature via the ohmic heating effect, determined by the surface current *I*_SD_^2^*R*^38^. Thus, when *V*_SD_ increases up to 50 V, we estimate that the local temperature of the graphene approaches 600 K.

**Fig. 2:**
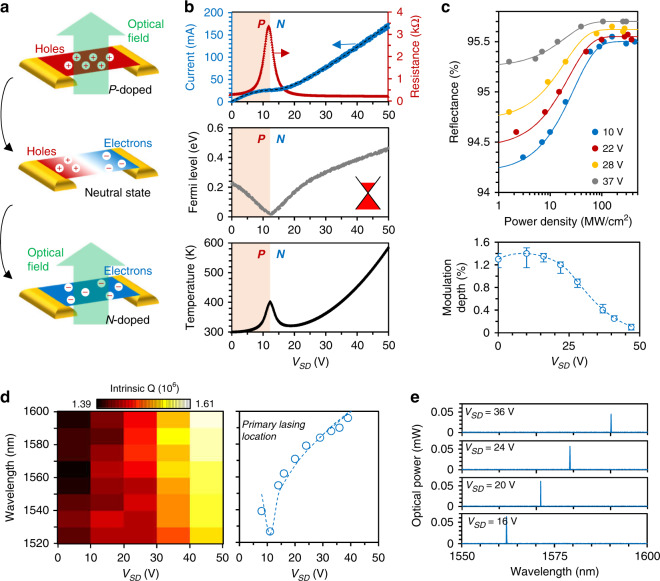
Optoelectronic tunability of the GFPR. **a** Schematic diagram showing that carrier doping tunes the optical transparency of graphene. **b** Electrical measurement of the graphene heterostructure. Top panel: *I*–*V* response and resistance tunability. Middle panel: the driving voltage changes the Fermi level of graphene from 0 to 0.45 eV across the Dirac point. Bottom panel: the correlation of *V*_SD_ and temperature in graphene, determined by the ohmic heating effect. **c** Saturable absorption of the graphene heterostructure, relying on the driving voltage. The modulation depth of the monolayer graphene decreases from ≈1.4 to ≈0.1%. **d** Measured *Q* factors *vs* wavelength, varying with *V*_SD_ from 0 to 50 V. Correspondingly, the primary laser wavelengths redshifts when increasing the driving voltage. **e** Spectra of the cw primary laser lines (measured with a pump power of 92 mW), which varies with the *V*_SD_

Figure 2c presents the nonlinear optical reflections of the in-fibre graphene. The ratio is normalized by the transmission of the dielectric Bragg reflector without graphene coverage (the setup is shown in Supplementary Section 3). The measured results are fitted by using the model *T(I)* = *1* − *ΔTexp(*−*I/I*_sat_*)* − *T*_ns_. Here, *T(I)* is the transmission intensity, *I* is the launched-in power density, *I*_sat_ is the saturation threshold, *T*_ns_ is the non-saturated transmission, and *ΔT* is the modulation depth. Determined by the linear Dirac corn of graphene, in which *|E*_F_| > *ħω*/2, the interband photon-electron transition will be dramatically blocked. Hence, when *V*_SD_ ≈ 10 V (close to neutral), the heterogeneous graphene device has the largest *ΔT* = 1.4%. Any external doping will decrease the *ΔT*. When the *V*_SD_ approaches 47 V, *ΔT* is only 0.1%. For the laser mode-locking operation, a relatively large *ΔT* is preferred. Actually, once the *V*_SD_ is higher than 37 V, it is difficult for the weak modulation depth to support a mode-locking state. A more detailed theoretical analysis of the tunable saturable absorption of graphene is discussed in Supplementary Fig. S1. As a result, the tuning of *ΔT* changes the total reflectance of the graphene deposited on the distributed Bragg reflector (DBR) facet, *R*_G_ = *R* *−* *ΔT*; here, *R* = 98% is the intrinsic reflectance.

As discussed above, the electrical tunability of the graphene on fibre enables us to effectively tune the intracavity resonance. Driven by the *V*_SD_, the surface current heats the graphene-deposited DBR mirror, thus changing the refractive index *n*_DBR_ and the grating period *Λ*_DBR_. This change causes a shift in the central wavelength of the graphene-deposited DBR mirror and induces an optical reflection imbalance in the spectrum, as determined by the grating model *λ* = *2n*_DBR_*Λ*_DBR_^39^. The temperature-induced *Λ*_DBR_ shift is at the 8 × 10^−^^4^ level, leading to an optical reflection imbalance in the spectrum (the measured spectral shift is shown in Supplementary Fig. S10). As a result, the wavelength-dependent *Q*-factor may be spectrally tunable. By using the fast laser scan technique, we measured the *Q*-factor distribution ranging from 1520 to 1600 nm. A high *V*_SD_ results in low graphene-based absorption; thus, the *Q*-factor increases overall. In addition, there is another typical trend. The spectral location of the highest *Q*-factor redshifts, as Fig. 2d illustrates. Supplementary Table S4 shows the numbers in more detail. Such a modification changes the primary lasing location in the spectrum. The primary lasing wavelength can be tuned from 1524 to 1591 nm, across almost the entire *C* + *L* band, and is only limited by the active band of the erbium gain. Here, the dots show the measured numbers, while the dashed curve plots the root-mean-square fitting, corresponding to the graphene electronics. Figure 2e plots four primary lasing lines, e.g., with a fixed pump power of 92 mW and *V*_SD_ = 16, 20, 24, and 36 V.

Correspondingly, when the pump power is 520 mW (higher than the mode-locking threshold), these primary laser lines evolve to varied mode-locking envelopes, as shown in Fig. 3a. When the *V*_SD_ varies from 16 to 36 V, the central wavelength of the mode-locking state is also tunable, from 1562 to 1594 nm. In addition to tuning the wavelength, we can also change the mode locking from the fundamental state to harmonic states by optimizing the pump power and delicately controlling the *V*_SD_. Due to the Kerr nonlinearity, harmonic-mode locking operates when the intracavity optical energy is high enough to split a single pulse circulating in the cavity into several pulses^40^. To achieve a harmonic state in this GFPR, we can increase the *V*_SD_ to decrease the graphene absorption or directly boost the pumping power. For instance, we show the fifth-order harmonic-locking state with a repetition rate ≈ 50 GHz (*V*_SD_ = 27 V, pump power 570 mW) and the eighth-order harmonic-locking state with a repetition rate ≈80 GHz (*V*_SD_ = 31 V, pump power 610 mW), in Fig. 3b. Limited by the bandwidth of the photodetector (PD), it is difficult to demonstrate such fast pulse trains in an oscilloscope. Instead, we show the SNR by using laser heterodyne beating (middle panels). This implementation is shown in Supplementary Fig. S11. This harmonic locking has an SNR > 40 dB. Related to the intrinsic-mode locking, the radio frequency (RF) SNR > 60 dB, such deterioration is due to super-mode noise. To suppress the super-mode noise in the GFPR, one can promote the saturable absorption threshold by carefully decreasing the *V*_SD_ (Supplementary Fig. S14). We also illustrate the frequency-resolved second-harmonic autocorrelation maps in the right panels. The fifth-order harmonic-locking and eighth-order harmonic-locking states have ~5% and 10% direct current bases, respectively. The single pulse duration here is at the single picosecond level.

**Fig. 3:**
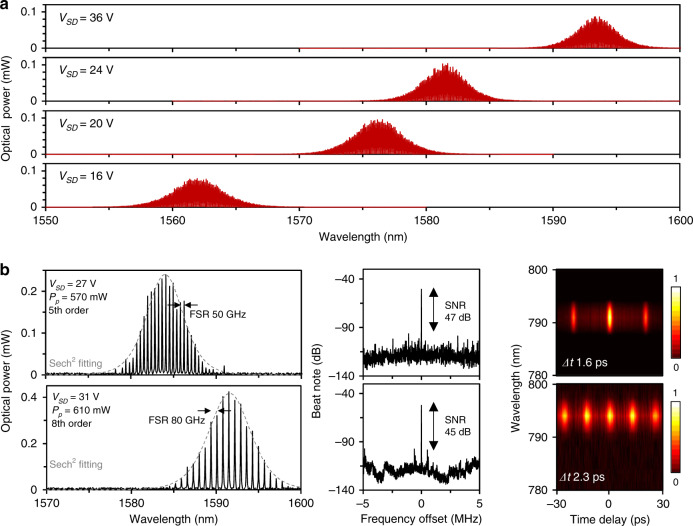
Electrical tunability of the mode-locking states. **a** Spectra of the fundamental mode-locked states (measured with a pump power of 520 mW). Increasing the *V*_SD_ enables the central wavelength of the laser envelope to redshift from 1562 to 1594 nm. **b** Examples of high-order harmonic-mode locking with repetition rates of 50 and 80 GHz. Panels from left to right: the optical spectra, the laser beat notes, and the frequency-resolved second-harmonic autocorrelation maps

The all-fibre structure can be conveniently connected afterwards to an optic fibre system for further amplification and supercontinuum broadening^41^. The experimental setup is shown in Supplementary Fig. S12. This system can span the electrically tunable mode-locked laser to be a wide frequency comb, spanning from 1300 to 2000 nm, which is over half an octave. Figure 4a plots the supercontinuum frequency combs, keeping line-to-line spaces. From top to bottom, we show the fundamental locking state with a repetition of 10 GHz, the fifth-order harmonic-locking state with a repetition of 50 GHz and the eighth-order harmonic-locking state with a repetition of 80 GHz. This amplified comb span is much wider than a typical soliton comb in silica microresonators^11^. By further optimizing the cavity dispersion and combining state-of-the art techniques such as chaos assistance^7^, it is also possible to achieve a broader comb span. However, in the amplifying and broadening process, noise is also amplified; thus, we need to stabilize the supercontinuum frequency comb.

**Fig. 4:**
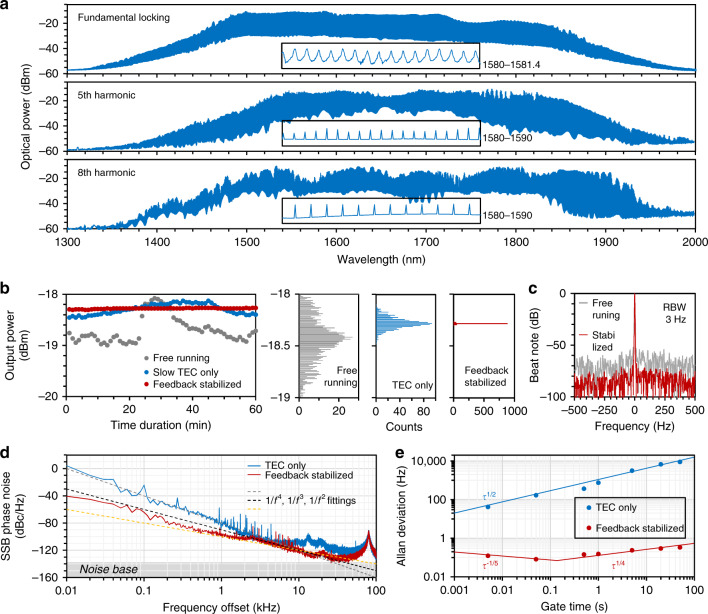
Broadening and stabilizing the laser combs. **a** Optical spectra of the locked combs via amplification and nonlinear spanning. Insets show the magnified structures. **b** Optoelectronic feedback on graphene with the TEC suppressing the power uncertainty from ±0.5 (free running) to ±0.01 dB (feedback stabilized). The statistics of 1000 points over 100 min show that the power variance decreases from 6.3 × 10^−2^ (free running) to 10^−4^ dBm^2^ (stabilized). **c** RF spectrum of the beat note before (grey) and after (red) stabilization, with a 3 Hz RBW. **d** Phase noise spectra. Locked on the RF synthesizer, *S(f)* is suppressed down to −120 dBc Hz^−1^, suggesting a timing jitter of 2.5 × 10^−15^ s. Here, the noise base is approximately −140 dB. **e** Allan deviation of the laser comb spacing. For long-term stability, the in situ feedback can provide four orders of optimization

In this unique graphene-fibre implementation, the graphene *p*−*n* junction provides a powerful means for direct laser stabilization via negative feedback. Similar to other microcavities, such as microdisks and microrings^42,43^, uncertainty in the spacing of the laser comb lines, or the FSR = *c*/2*n*_eff_*L*, mainly comes from the thermal instability of the effective refractive index *n*_eff_, where *c* is the light velocity in vacuum and *L* is the cavity length. In addition, the intracavity power fluctuation also influences the jitter noise via Kerr nonlinearity^44^. To compensate for the dynamic noise, a thermal electric cooler (TEC) with a millisecond response is not fast enough for the resonator with a 10 GHz pulse operation. In previous studies, electrical feedback was commonly compensated at the pump^14^. In the GFPR, we build a feedback loop to control the cavity directly, taking advantage of the fast optoelectronic response of graphene. A modulation bandwidth larger than 200 MHz is shown in Supplementary Fig. S15, while the feedback schematic diagram is shown in Supplementary Fig. S11.

A stable RF synthesizer is used for locking the *f*_FSR_ of the laser, then the beat note of the laser and the RF synthesizer is sent to loop filter, and finally to an electric amplifier. This process is similar to a lock-in amplifier. Afterwards, the amplified voltage is negatively added to the GPFR to compensate for the *V*_SD_. Figure 4b plots the stabilization of the laser comb power for the fundamental mode-locking operation. The power is monitored on-line by using a 1% coupler and a tunable attenuator. The TEC suppresses the intensity noise from free running ±0.5 to ±0.1 dB and the TEC + RF feedback double stabilization further enables the power uncertainty down to ±0.01 dB; this number is only limited by the resolution of the power metre. By counting a thousand points in 100 min, we also show the statistical histogram distributions. The detected power is kept at −18.28 dBm, with an estimated variance <10^−4^ dBm^2^. By mixing with a stable diode laser reference, Fig. 4c shows the beat note of the comb. Before stabilization, the SNR is ≈50 dB (the broadening also introduces additional noise). After stabilization, the beat note illustrates an SNR > 70 dB, with a linewidth only limited by the electrical spectrum analyzer (ESA) resolution of 3 Hz.

The measured single sideband (SSB) phase noise spectra *S(f)* are shown in Fig. 4d. For the state without feedback stabilization (only the TEC works), the phase noise is −80 dBc Hz^−1^ at 1 kHz. At low frequencies (10 Hz to 2 kHz), the phase noise roughly obeys the *f*^−4^ dependence on the offset frequency in the vicinity of the carrier. This close-to-carrier behaviour suggests that the phase noise is now dominated by the random-walk frequency (40 dB/decade), rather than the quantum noise phase diffusion. Once locked by the RF synthesizer, the mode-locked laser comb with a 10 GHz repetition demonstrates an *S(f)* lower than −90 dBc Hz^−1^ at 1 kHz and even reaches −120 dBc Hz^−1^ at 10 kHz, which is greatly limited by the instrument. Such noise suppression is comparable to high spectral purity Kerr solions in silica and MgF_2_ microcavities^8,45^. Below 1 kHz, the feedback stabilizes *S(f)* and demonstrates a *f*^−2.7^ dependence on the offset frequency; this is mainly determined by a mixture of flicker frequency noise and white frequency noise from the RF synthesizer. In addition, the thermal noise oscillation at ~80 kHz comes from the measurement system, such as the optical amplifier. Referring to the timing jitter *τ*_J_ = *∫S(f)*/*(*2*πR)*, wherein *R* is the repetition rate, we estimate the temporal instability per roundtrip down to 2.5 × 10^−15^ s. Figure 4e plots the Allan deviation *σ*_A_*(τ)* of the *Δf*_FSR_ before and after feedback stabilization. Here, *τ* is the gate time. After down mixing, the beat frequency is counted with a Λ-type frequency counter. In the free running state, *σ*_A_*(τ)* increases from 41 to 8724 Hz, approximately meeting the *τ*^1/2^ correlation. This result also verifies that the free-running laser comb is limited by technical noise. The optoelectronic feedback improves the laser comb in long-term operation, with a minimum *σ*_A_*(τ)* below 0.1 Hz when the gate time is approximately 0.2 s. First, *σ*_A_*(τ)* decreases with the trend *τ*^−1/5^ and then increases back to the initial value with a slow increasing slope of *τ*^1/4^. This in situ feedback scheme of the graphene-fibre resonator is also applicable for other repetition rates.

Leveraging the electrical tunability of the heterogeneous graphene device incorporated in a fibre microresonator, we demonstrate its capability to control laser microcomb dynamics in situ. Taking advantage of the tunnelling diode effect, we realize remarkable graphene Dirac fermion tuning from 0 to 0.45 eV. This result leads to a controllable modulation depth in the range of 0.1 to 1.4%. Consequently, mode-locked laser frequency combs with unprecedented dynamic tunability are demonstrated. Moreover, the graphene-integrated microlaser device provides a powerful way to opto-electrically stabilize the comb lines after supercontinuum amplification and the SSB phase noise reaches the instrument-limiting floor of −120 dBc Hz^−1^ at a 10 kHz offset, thereby suggesting a timing jitter of less than 2.5 × 10^−15^ s per roundtrip. The broadened comb envelopes span over half an octave, which is sufficient for 2*f*–3*f* self-referencing. Such realization of the dynamic control and stabilization of the microcomb in a heterogeneous graphene-fibre microcavity provides a new platform for the interfacing of single-atomic-layer optoelectronics and ultrafast photonics, and will promote versatile applications for arbitrary waveform generation, fibre communication, signal processing, and spectroscopic metrology.

## Methods

### Theoretical analysis

(1) Electrical modulation determines the saturable absorption of graphene. The surface carrier density of graphene relies on the *V*_SD_ via the tunnelling diode effect. Determined by kinetic equations, this alteration dramatically influences the Pauli blocking in graphene. (2) Surface current enables thermal engineering. In the graphene, the surface current in the graphene considerably increases the surrounding temperature, leading to an unbalanced reflection with the DBRs. (3) Tunable intracavity energy manipulates laser operations. Once (*PT*_R_)^2^ > *E*_N_*E*_g_*ΔT*, stable-mode locking without amplitude fluctuation can be realized. Here, *T*_R_ is the roundtrip time, *P* is the laser power, *E*_N_ and *E*_g_ are the saturation energies of the gain media and graphene, respectively, and *ΔT* is the modulation depth. (4) Active stabilization. The fast optoelectronic response of the graphene device can suppress the noise related to random-walk frequency, flicker frequency, white frequency, flicker phase, and white phase. Details are discussed in Supplementary Section 1.

### Device design and fabrication

The erbium/ytterbium-doped single-mode fibre section (EYDF, Nufern) offers a 980 nm/1550 nm pump-gain efficiency > 30% m^−1^. The insertion loss of the EYDF and standard SMF-28e is <0.12 dB. All fibre sections were carefully cut and capsuled in a ceramic adapter. Before further processing, each fibre end of the cavity was polished to mirror smoothness, forming the *F*–*P* resonator. Multilayer dielectric reflective film-based DBRs (SiO_2_ + Ta_2_O_5_) were coated on both sides of the *F*–*P* cavity. The reflectance of each DBR was higher than 98%. Monolayer graphene on copper foils was grown by the CVD method. A layer of a poly(methyl methacrylate) (PMMA) protection film was applied by spin coating (3000 r min^−1^ for 30 s) on the surface of the foil and baked at 150 °C. Then, a free-standing PMMA-supported graphene film was obtained by etching the bottom copper foil with FeCl_3_ solution and wet transferred onto the surface of the fibre section. Then, the PMMA was dissolved in acetone. A customer-designed soft stencil lithograph made it physically aligned and was transferred on top of the erbium/ytterbium-doped high *Q*-factor fibre Fabry–Perot resonator. Finally, Ti/Au contact electrodes (5 nm/50 nm) were fabricated through e-beam evaporation. More information about the nano-fabrication and material characterizations are shown in Supplementary Section 2.

### Experimental setups and optoelectronic implementations

For optical transmission spectrum measurements, we used a tunable laser (Agilent 8168A, USA) with a stable CW output (typical linewidth 300 kHz) scanning from 1520 to 1610 nm. The output power of the tunable laser was fixed at 1 mW to avoid nonlinear excitations. The spectral transmissions were detected by an infrared band PD (Thorlabs DET08CFC, USA) and then plotted in a triggered oscilloscope with a 500 MHz bandwidth (Tektronix TDS3052C, USA). For saturable absorption determination, we used a high-power femtosecond laser (mode-locked fibre laser with a 37 MHz repetition rate and 500 fs pulse delay) to provide a sufficiently high peak power (maximum peak power of 2.2 kW, power density of 4.4 GW cm^−^^2^). The output power was detected by a high accuracy power metre. For laser generation and control, we used a single-mode 980 nm LD as the optical pump (maximum power of 1.5 W). A high-power 980/1550 wave division multiplexer with a bandwidth of 1520 to 1610 nm divided the pump and laser. The GFPR was fixed on a V-groove fixture with a TEC (Thorlabs TC 200, resolution 10 mK, USA). The dynamics of the laser combs could be monitored by using an optical spectrum analyser (Yokogawa 6370D and 6375B, Japan), an oscilloscope, and a 2 ~ 43.5 GHz RF-ESA (Rohde & Schwarz, Germany). The setups are specifically shown in Supplementary Section 3.

### Supercontinuum amplification and feedback stabilization

We used erbium doped fibre amplifiers to amplify the comb pulses. Then, a section of dispersion-shifted highly nonlinear fibre with a nonlinear coefficient of 0.04 Wm^−1^ was used to broaden the mode-locked combs from 5 (3 dB span) to over 200 nm (3 dB span). Then, we extracted the intrinsic beat frequency (*f*_FSR_) and locked it on an RF synthesizer via a loop filter and an electric amplifier. The output voltage of the loop filter was further amplified and then negatively added to the GPFR to compensate for the *V*_SD_. In this procedure, we kept the TEC on to obtain better performance. The implementation of this method is shown in Supplementary Section 3.

## Supplementary information

SUPPLEMENTARY INFORMATION

## Data Availability

The supporting data for the findings in this study are available from the corresponding author upon reasonable request.
