# Supplementary material for: Electrically controllable laser frequency combs in graphene-fibre microresonators
Source: Light Sci Appl. 2020 Nov 9;9:185. doi: 10.1038/s41377-020-00419-z (PMC7652939; doi:10.1038/s41377-020-00419-z)
Supplement: Supplementary file 1 — SUPPLEMENTARY INFORMATION [file 41377_2020_419_MOESM1_ESM.pdf]

Supplementary Information of

Electrically controllable laser frequency combs in graphene-fibre microresonators

Chenye Qin[1,6], Kunpeng Jia[2,3,6], Qianyuan Li[4,6], Teng Tan[1,5,6], Xiaohan Wang[2,3], Yanhong Guo[1], Shu-Wei Huang[3#], Yuan Liu[4#], Shining Zhu[2], Zhenda Xie[2]*, Yunjiang Rao[1,5]*, Baicheng Yao[1]*

[1]*Key Laboratory of Optical Fibre Sensing and Communications (Education Ministry of China), University of Electronic Science and Technology of China, Chengdu, China.*

[2]*National Laboratory of Solid State Microstructures and, School of Electronic Science and Engineering, School of Physics, and College of Engineering and Applied Sciences, Nanjing University, Nanjing, China.*

[3]*Department of Electrical, Computer, and Energy Engineering, University of Colorado Boulder, Boulder, CO 80309, United States*

[4]*Key Laboratory for Micro-Nano Optoelectronic Devices (Education Ministry of China), School of Physics and Electronics, Hunan University, Changsha, China.*

[5]*Research Centre for Optical Fibre Sensing, Zhejiang Laboratory, Hangzhou, China.*

[6]*These authors contributed equally.*

This Supplementary Information consists of the following sections:

S1. Concepts and theoretical analysis

S2. Device fabrication and properties.

S3. Setups and extended measurements.

Supplementary Figures S1-S15

Supplementary References S1-S42

S1. Concepts and theoretical analysis

Table S1 compares some typical compact mode locked lasers with high repetition rate reported previously, including fiber lasers, on-chip Kerr soliton combs and solid-state lasers. More specifically, **Table S2** compares some typical implementations by integrating graphene electrical device in fiber laser loop. This work is the first attempt to integrating graphene p-n junction in an in-fiber microresonator, which has the unique E-O tenability, and the best stability comparable to the fully-stabilized Kerr soliton combs. **Table S3** introduces some Kerr and EO soliton microcombs with similar repetition rate, this work shows comparable performance.

Table S1 | Comparison of the high repetition mode locked lasers

	**Mode locking scheme**	**Electrical controllability**	**Technical performances**				
			Intrinsic rep. rate	Pulse energy	Pulse duration	Beating linewidth	Phase noise
This work	*Graphene SA&soliton*	*Yes*	*10 GHz*	*50 pJ*	*710 fs*	*3 Hz*	*-120 dBc/Hz @10 kHz*
S1	*Graphene SA*	*No*	*10 GHz*	*NG*	*865 fs*	*NG*	*NG*
S2	*Stretched soliton*	*No*	*1.87 GHz*	*41 pJ*	*83 fs*	*NG*	*NG*
S3	*Graphene SA*	*No*	*38 MHz (typical for fiber loops)*	*190 pJ*	*910 fs*	*NG*	*NG*
S4	*Graphene SA*	*No*	*1.5 GHz*	*220 nJ Q-switching inside*	*1.06 ps*	*>5 MHz*	*NG*
S5	*Crystal SA*	*No*	*15 GHz*	*95 pJ*	*815 fs*	*NG*	*NG*
S6	*Crystal SA*	*No*	*9.6 GHz*	*NG*	*1.9 ps*	*6 kHz*	*> -70 dBc/Hz @10 kHz*
S7	*Quantum wall*	*No*	*40 GHz*	*NG*	*1 ps*	*25 kHz*	*-60 dBc/Hz @10 kHz*

**NG*: not given.

Table S2 | Comparison of the mode locked fiber lasers with graphene device intracavity

	**Spectral width**	**Pulse duration**	**Intrinsic Rep. rate**	**Wavelength tunability**	**FSR stabilized**
This work	> 150 nm after broadening	710 fs	10 GHz	Yes	Yes
S1	< 10 nm after broadening	865 fs	10 GHz	No	No
S8	8 nm	423 fs	30.9 MHz	No	No
S9	0.6 nm	NG	4 MHz	No	No
S10	1.82 nm	1.57 ps	4.35 MHz	No	No

**NG*: not given.

Table S3 | Performance comparison of the laser comb and Kerr and EO soliton microcombs with similar repetition rate

	Technical Scheme	Electrical controllability	Intrinsic Rep. rate	Spectral width	Pulse duration	Phase noise
This work	Graphene SA&soliton *in-fiber*	*Yes*	10 GHz	> 150 nm after broadening	710 fs	*-120 dBc/Hz @10 kHz*
S11	*Kerr soliton in-fiber*	*No*	9.77 GHz	80nm	137 fs	NG
S12	*Kerr soliton in-Si_3N_4* ring	*No*	17.9 GHz	>60nm	>100 fs	*-110 dBc/Hz @10 kHz*
S13	*Kerr soliton in-Silica*	*No*	16.4 GHz	> 1.1 μm after broadening	<200 fs	NG
S14	*Kerr soliton in-MgF_2*	*No*	10 GHz	30nm	NG	*-120 dBc/Hz @10 kHz*
S15	*Kerr soliton in-MgF_2*	*No*	12.5 GHz	30nm	220 fs	NG
S16	*Kerr soliton in-MgF_2*	*No*	14 GHz	> 10 nm	NG	*-135 dBc/Hz@10 kHz*
S17	*EO comb in $LiNbO_3$*	*Yes*	10.453 GHz	80nm	NG	NG
S18	*EO comb In $LiNbO_3$*	*Yes*	8.9 GHz	35nm	NG	NG

**NG*: not given.

Graphene optics relies on its complex conductivity $\sigma_g = \sigma_{g,r} - \sigma_{g,i}$, which is determined by the optical frequency f, quasi Fermi level E_F, carrier relaxation lifetime τ, and absolute temperature T [S19].

$$\sigma_g(f, E_F, \tau, T) = \frac{ie^2\left(2\pi f - \frac{i}{\tau}\right)}{\pi\hbar^2}\left\{\frac{1}{\left(2\pi f+\frac{i}{\tau}\right)^2}\int_0^\infty \varepsilon\left[\frac{\partial f_d(\epsilon)}{\partial\epsilon} - \frac{\partial f_d(-\epsilon)}{\partial\epsilon}\right]d\epsilon - \int_0^\infty\left[\frac{f_d(-\epsilon)-f_d(\epsilon)}{\left(2\pi f+\frac{i}{\tau}\right)^2-4\left(\frac{\epsilon}{\hbar}\right)^2}\right]d\epsilon\right\} \quad \text{(S1)}$$

In this equation, $\tau \approx 10^{-13}$ s level for graphene material, the electronic energy of graphene ϵ obeys the Fermi-Dirac distribution

$$f_d(\epsilon) = \left[e^{\frac{\epsilon - E_F}{k_B T}} + 1\right]^{-1} \quad \text{(S2)}$$

Here $\hbar$ = 1.05×10^{-34} eV·s is the reduced Planck constant, k_B = 1.3806505×$10^{-23}$$J$/K is the Boltzmann's constant, and e = -1.6×$10^{-19}$$C$ is the unit charge. The surface carrier density of graphene relies on the carrier density (either electrons or holes), which can influence the Pauli blocking in graphene dramatically, driven by the Kinetic equations [S20, S21, S22]. The macroscopic surface current $\boldsymbol{J(t)}$ is a integration over every transition electron with momentum $\boldsymbol{k}$,

$$\boldsymbol{J}(t) = \frac{g_s g_v}{4\pi^2}\int \boldsymbol{j_k}(t)d^2\boldsymbol{k} \quad \text{(S3)}$$

Here $g_s = g_v = 2$ presents for spin and valley degeneracies. ***J(t)*** could be separated as intraband and interband components, also corresponding to the intraband and interband conductivity respectively. For graphene saturable absorptions at C + L band, the photon energy is around 0.4 eV, hence the interband transition contributes majorly. The interband current density $\boldsymbol{J}_{inter}(t)$ is then written as

$$\boldsymbol{J}_{\boldsymbol{inter}}(t) = \frac{2ev_F}{\pi^2} Re\{ie^{-i2\pi ft} \int sin\varphi(\Gamma_{\boldsymbol{k}}^{-} - \Gamma_{\boldsymbol{k}}^{+*})d^2\boldsymbol{k}\} \quad \text{(S4)}$$

Here $\Gamma_{\boldsymbol{k}}^{-}$- $\Gamma_{\boldsymbol{k}}^{+*}$ is the conjugate coefficient of the carrier density determined by both the Fermi level and the local temperature, f is the optical frequency, φ relates to phase information. In consequence, total interband optical absorption is

$$\alpha_{inter} = \alpha_{ns}(f_v - f_c) = \frac{\int_{-\frac{1}{2f}}^{\frac{1}{2f}} \boldsymbol{J}_{inter}\cdot\boldsymbol{E}(t)dt}{\sqrt{P(f)}/f} \quad \text{(S5)}$$

Here α_{ns} the absorption coefficient with a full valance band (for example T =0 K), f_v and f_c are the Fermi occupation probabilities for the populations in the valance and conduction bands, obeying the dispersion formula of $f_d(\epsilon)$. ***E(t)*** is the electron energy, $P(f)$ is the optical power. As a result, the correlation of 'power density *vs* normalized transmission' can be approximately written as

$$T(I) = 1 - \alpha_{inter} = 1 - \Delta T \exp\left(-\frac{I}{I_{sat}}\right) - T_{ns} \quad \text{(S6)}$$

Here $I=P(f)/A_{eff}$ is the power density, ΔT is the modulation depth, I_{sat} is the saturation threshold related to the graphene relaxation time τ, T_{ns} is the non-saturated transmission, determined by the fine structure constant. When temperature increased, due to the hot carrier-phonon interactions [S23, S24], the graphene relaxation time τ increases from tens of femtoseconds to sub picosecond. Consequently, a higher temperature can induce a lower ΔT in total, a higher I_{sat}, and a higher T_{ns}.

Such a tunability of nonlinear absorption is achievable by carrier injection, such as adding an external electrical field or a gate voltage in a FET structure [S20, S25]: More electrons distribute in the valance band, the efficiency of the photon-electron transition would be suppressed. Besides, such a carrier density modification can be implemented by tuning the surface current density directly, in the graphene p-n junction structure [S26]. For a monolayer graphene, quasi Fermi level $|E_F|$ is proportional to the carrier density, relying on the relationship [S27]

$$|E_F| = \hbar|v_F|\sqrt{\pi N} \quad \text{(S7)}$$

Here N is the carrier density, while v_F indicates the Fermi velocity, which is sensitive to impurity doping and temperature. **Figure S1a** maps the calculated Dirac Fermion tunability of the atomic graphene, by tuning N continuously. For simplification, here we fix the mobility of graphene at room temperature 10^4 $cm^2V^{-1}s^{-1}$, and $v_F = 10^6$ ms^{-1}. In the meantime, this reduces the linear loss, which is useful for keeping the laser cavity Q factor. By fixing $f = 189.8$ THz (wavelength 1580 nm, or photon energy 0.785 eV), **Figure S1b** simulates the saturable absorption properties of the monolayer graphene absorber, with adding external carriers. When layer number is smaller, the normalized modulation efficiency could be higher [S28]. In details, **Figure S1c** and **S1d** conclude the Fermi level dependent ΔT and I_{sat}, in calculations. In practice, the intracavity optical power may be limited, and the intraband absorptions also contribute, the total modulation efficiency would be lower.

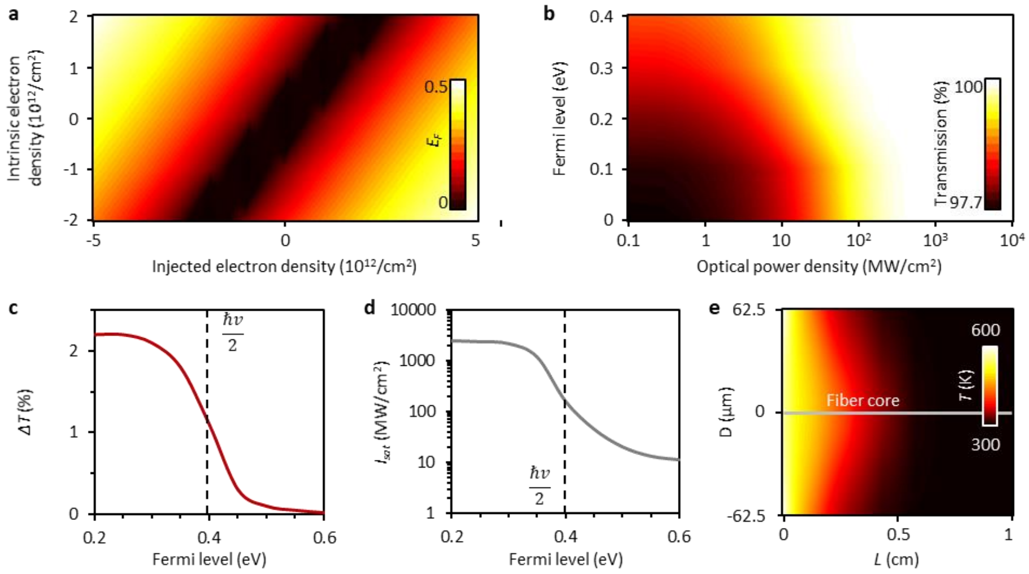

Figure S1 | Electrical tunability of graphene intracavity. a, Dirac Fermion distribution of graphene, relying on the carrier density. **b,** Saturable absorption map of the monolayer layer graphene sample. The intrinsic absorption for each monolayer graphene is $\pi\alpha$. **c** and **d,** Fermi level dependent ΔT and I_{sat}. Due to the Pauli blocking physics, a higher carrier density brings lower modulation depth, lower saturation threshold, while higher total optical transmission. **e,** Simulated result of the thermal field distribution in the fiber.

The surface current in the graphene raises the surrounding temperature considerably, according to the *Ohm's* theorem. This influences both the reflector and the cavity. The cavity composes a pair of

multilayer dielectric Bragg gratings as the reflectors. Central reflection wavelength of a Bragg grating λ_R relies on

$$\lambda_R = 2n_{eff}\Lambda \tag{S8}$$

Here n_{eff} is the mode effective index of the transmitting light, Λ is the grating period. Reflection linewidth is determined by the period number [S29]. For a silica based F-P resonator with thermal RI sensitivity sub 10^{-5} per Kelvin [S30], a 300 K scale thermal tuning can change the n_{eff} in 10^{-3} level, enabling resonance to shift in sub GHz. For the double FBG cavity architecture, the resonance region should obey the $R1*R2$ spectrally. Once one of the two reflectors changes, e.g. by the hot graphene layer, the reflection spectrum could be modulated. As a result, the central wavelength of $R1*R2$ shifts, enabling the tunability of the laser wavelength. By using the finite element method, **Figure S1e** simulates the thermal distribution of the F-P resonator, which is packaged in a standard ceramic core, when the graphene temperature is 600 K, located at the left side of the cavity. In kinetics, the operation of the graphene based F-P fiber laser could described by using the temporal rate equations [S31, S32]

$$\begin{aligned} T_R\frac{\partial P}{\partial t} &= 2(g-l-\alpha)P \\ \frac{\partial g}{\partial t} &= -\frac{g-g_0}{\tau_N}-\frac{gP}{E_N} \\ \frac{\partial \alpha}{\partial t} &= -\frac{\alpha-\alpha_0}{\tau_g}-\frac{\alpha P}{E_g} \end{aligned} \tag{S9}$$

Here T_R is the roundtrip time, P is the laser power, g the intracavity gain per round-trip, l the cavity loss, α the absorption of the graphene in each round-trip, g_0 the small signal gain per roundtrip, α_0 the unsaturated but saturable loss per round-trip, τ_N and τ_g the lifetimes of the gain media and the absorption recovery time of graphene respectively, while E_N and E_g are the saturation energies of the gain media and graphene respectively, also influenced by the τ_N and the τ_g. When $g\text{-}l\text{-}\alpha > 0$, lasing occurs. For the graphene-based absorption, α is determined by the instantaneous laser power, following $\alpha = \alpha_0/(1+\tau_g P/E_g)$. If $\partial\alpha/\partial t \neq 0$, $\partial P/\partial t \neq 0$ (the output is discrete pulses), which could be induced by either Q-switching or mode locking. For stable mode locking without amplitude fluctuation, i.e. $\partial g/\partial t = 0$, there is a criterion $(PT_R)^2 > E_N E_g \Delta T$. Accordingly, by increasing the intracavity optical power, the laser state could be tunable from CW operation, multimode operation, to mode locking and harmonic mode locking operation, as shown in **Figure S2a** schematically. In previous literatures for a fiber loop laser, such a tuning could be achieved via changing the pump power [S33]. For the graphene based

semiconductors or heterostructures, these states can be achieved by changing the saturable absorber electrically [S10]. By using the FDTD calculation, **Figure S2b** to **S2e** simulates the potential temporal evolutions of the 10 GHz FSR Er^{3+}/Yb^{3+} doped fiber resonator with total loss 1%+ΔT, with the same initial pump power 500 mW, and gain efficiency 8% per roundtrip.

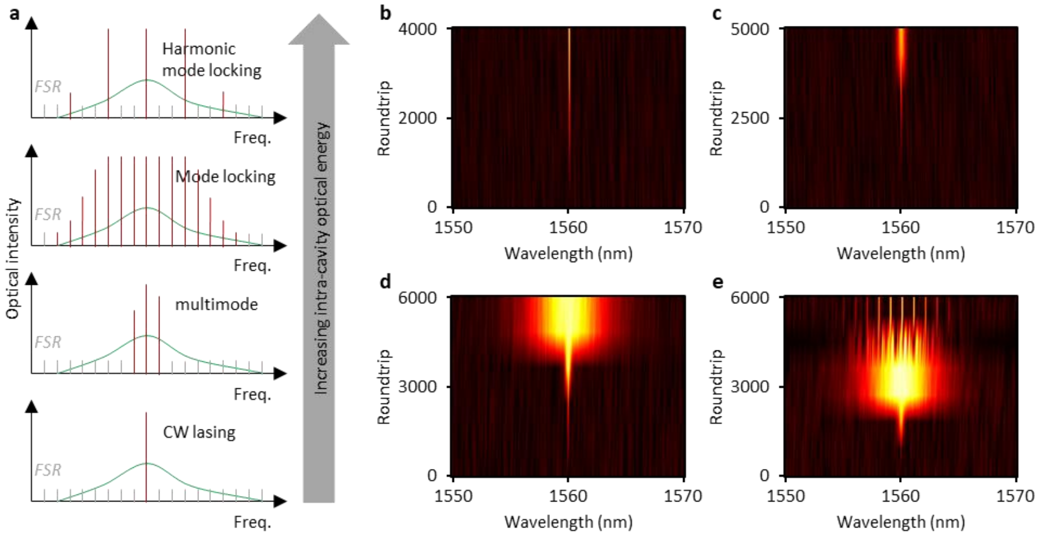

Figure S2 | Spectral evolutions. a, Schematic laser dynamics. **b,** For $\Delta T = 0.5\%$, I_{sat} = 100 MWcm^{-2}, due to the low modulation depth, the laser is CW. **c,** For $\Delta T = 6\%$, I_{sat} = 100 MWcm^{-2}, the intracavity energy accumulation is slow, it can output multimode state. **d,** For For $\Delta T = 1\%$, I_{sat} = 10 MWcm^{-2}, the laser can be mode locked. **e,** For $\Delta T = 1\%$, I_{sat} = 1 MWcm^{-2}, harmonic mode locking operates.

The mode locking in the Fabry-Perot (F-P) resonator is determined by the longitude mode selection driven by the graphene. The saturable absorption selects high power resonance out, and then due to the intra-cavity third order nonlinearity of the fiber, self-phase-modulation and modulation instability induce spectral broadening during the pump-lasing process [S34], leading to further mode competition in the laser cavity. Related to the Fourier's transform, in time domain, the resonant mode families would be phase-locked via dissipative soliton generation [S35]. Once the high power pulses generated by modulation instability satisfies the ultrafast phase matching:

$$\Delta k_D(f) + \Delta k_{NL}(f) \approx \beta_2 (FSR)^2 + \frac{2\pi f n_{NL}(f)}{cA_{eff}} P(f) = 0 \qquad \text{(S10)}$$

In above equation, $\Delta k_D(f)$ and $\Delta k_{NL}(f)$ indicates the phase shift from the anomalous dispersion and from the intra-cavity nonlinearity at specific frequency f respectively, β_2 is the average group-velocity-

dispersion (GVD) of the cavity, $n_{NL}(f)$ is the nonlinear refractive index, typically ≈ 3×10^{-20} m^2W^{-1} in fiber, A_{eff} is the effective mode area, ≈ 50 μm^2 for our HE_{11} dominant fiber system.

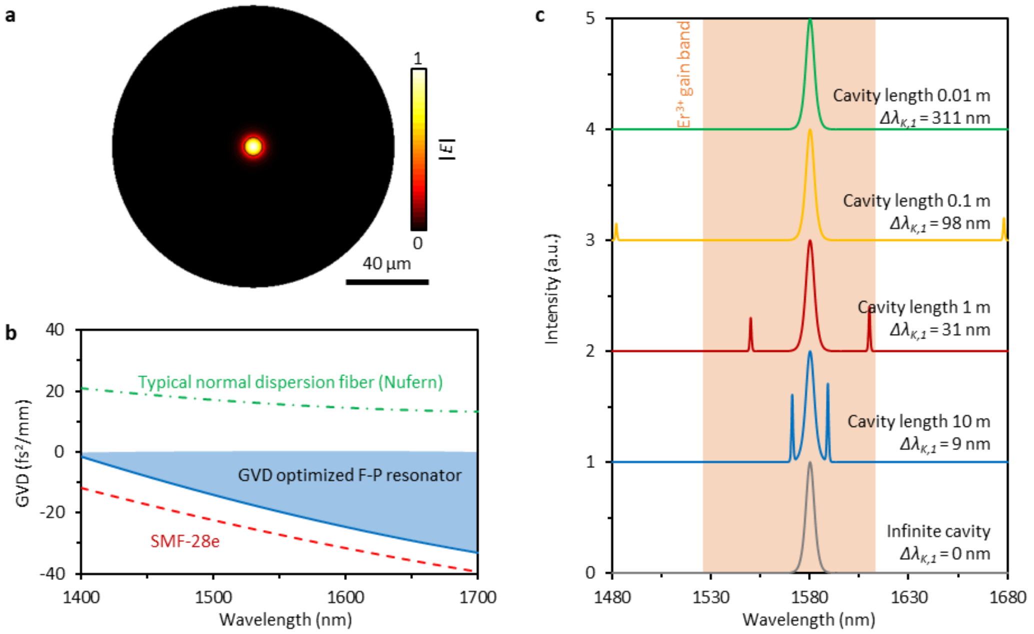

Figure S3 | Er^{3+}/Yb^{3+} doped F-P resonator for soliton mode locking. a, Simulated electric field intensity distribution of the fundamental mode in fiber (Sectional view). **b,** Chromatic dispersion design, the green curve, red curve and blue curve shows the β_2 of typical normal dispersion fiber, the single mode fiber and our GVD optimized Er^{3+}/Yb^{3+} resonator, respectively. **c,** Calculated mode locking laser spectra considering the Kelly bands, for varied cavity length from 10 m to 0.01 m.

Figure S3a shows the simulated electric field of a typical Er^{3+} / Yb^{3+} doped fiber. In our laser cavity, β_2 is fixed in a laser cavity, the soliton state is achieved by using graphene tunable saturable absorber via controlling the intra-cavity pulse power $P(f)$ [S36]. To ensure the phase matching, GVD of the F-P cavity were optimized flatten and anomalous, as shown in **Figure S3b**, total timing delay induced by the GVD in a roundtrip is -0.0004 ps^2. In the GVD managed F-P resonator, phase shifts induced by the nonlinearity and the single mode fiber are discrete in one roundtrip, this minor periodic disturbance enables the dispersive wave propagation and the Kelly band in spectrum [S37, S38],

$$\Delta\lambda_{K,N} = \pm N\lambda_0\sqrt{\frac{2N}{cDL} - 0.0787\,\frac{{\lambda_0}^2}{(c\tau_p)^2}} \qquad \text{(S11)}$$

Here λ_0 is the central wavelength, $D = -2\pi c/\lambda^2$ is the GVD parameter, N is the sideband order, L is the fiber cavity length, τ_p is the pulse duration, obeying the Fourier transform limit $\Delta f\tau_p = 0.315$ for

sech^2 pulses. For example, a central wavelength 1580 nm soliton mode locked laser with GVD parameter 15.7 ps km^{-1}nm^{-1}, τ_p = 500 fs, **Figure S3c** simulates the first order Kelly band distributions in spectrum, for cavity length 0.01 m, 0.1 m,1m and 10 m. The $\Delta\lambda_{K,1}$ are 311 nm, 98 nm, 31 nm and 9 nm, respectively. In experiment, because the miniature F-P resonator is very short, we cannot see the Kelly band in the lasing region.

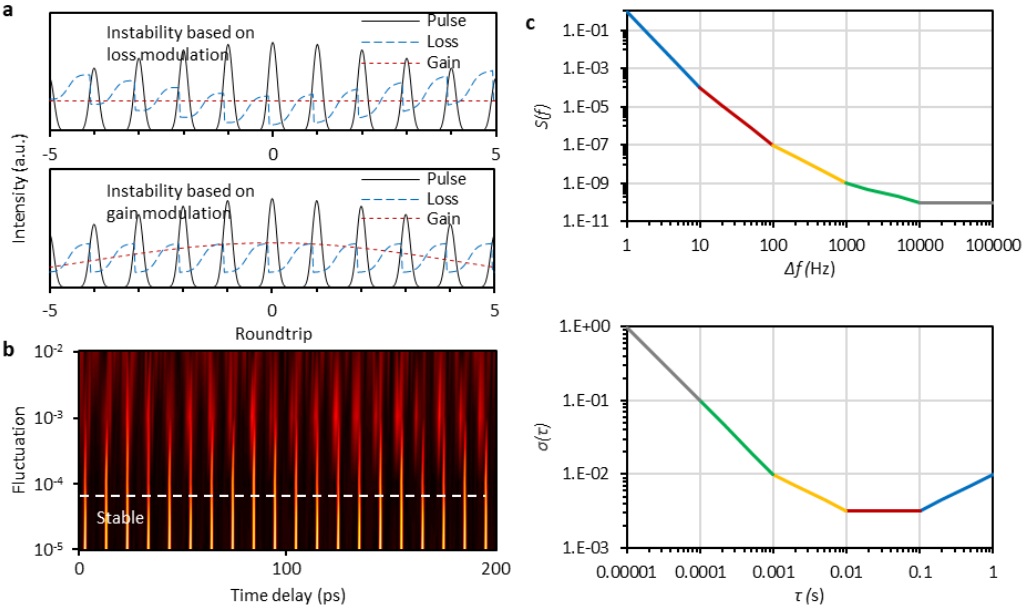

Figure S4 | Simulated spectral evolutions. a, Schematic demonstration of the intensity instabilities. **b,** Simulated FSR fluctuation, from 10^{-4} to 10^{-2}. **c,** Relationship of the phase noise and the Allan deviation.

Instabilities for the F-P laser are induced by intensity noise, intracavity phase noise, and mode competition majorly. First, the intensity noise is due to the potential instabilities from either the saturable absorber or the gain media. For a weak P, PT_R has to be accumulated to satisfy the E_g, thus Q-switching envelop appears. On the other hand, once P is over high, due to the Kerr self-focusing induced spatial hole-burning [S39], E_N turns to be even higher, also impedes the stabilization. **Figure S4a** demonstrates the possible cases schematically. Although P might be unstable, the graphene device with tunable ΔT provides a good means to stabilize the mode locking, by using a feedback loop.

Second, we consider the phase noise, which can be induced by the FSR instability intracavity. For a F-P cavity with 10^6 Q factor in C/L band, the FSR is only around 120 MHz, sub GHz instability is

enough to damp the locking state. **Figure S4b** maps the simulated temporal response of a 10 GHz resonator with linewidth 100 MHz, when *FSR* fluctuation increases from 10^{-4} to 10^{-2}. Typically, there are five types of noises, dependent on random walk frequency, flicker frequency, white frequency, flicker phase and white phase [S40]. We show their frequency-time relationship in **Table S4**. We plot a typical frequency offset – phase noise correlation and the corresponding Allan deviation containing all the noises in **Figure S4c** and **S4d**. Here the unit is normalized. Here the Allan deviation is estimated in the following equation. Here τ is the gate time, y is the fractional frequency, M is the number of sample.

$$\sigma_A(\tau) = \sqrt{\frac{1}{M}\sum_{k=1}^{M}\frac{(\bar{y}_{i+1}-\bar{y}_i)^2}{2}} \quad \text{(S12)}$$

Table S4 | Possible noises intracavity

	S(f)	**σ(τ)**
Random walk frequency	f^{-4}	$\tau^{1/2}$
Flicker frequency	f^{-3}	τ^{0}
White frequency	f^{-2}	$\tau^{-1/2}$
Flicker phase	f^{-1}	τ^{-1}
White phase	f^{0}	τ^{-1}

In above analysis, we assume the temperature distribution in the graphene sheet is uniform. However, because the intracavity graphene is illuminated by the high power laser, photo heat may induce Seebeck effect [S41], which is also an influence for the carrier density distribution in the graphene. In some reports, such thermal effect also shows tunability on nonlinear absorption [S9]. **Figure S5a** maps the energy integration of $f_d(\epsilon)$ varying with the temperature T. For a higher temperature, more electrons distribute in the conduction band. For the pristine graphene, when T =0 K, f_v - f_c = 1, all the valance band is filled with electrons, it could have the highest modulation depth in principle. Although for any state that $0 < f_v - f_c < 1$, Pauli blocking is possible [S28], at a high temperature, there are more electrons in the valence band, the photoelectron transition efficiency could be suppressed. **Figure S5b** plots the calculated possibility f_v - f_c, while **Figure S5c** plots the ΔT of a ten layered graphene sample, depending on surface temperature.

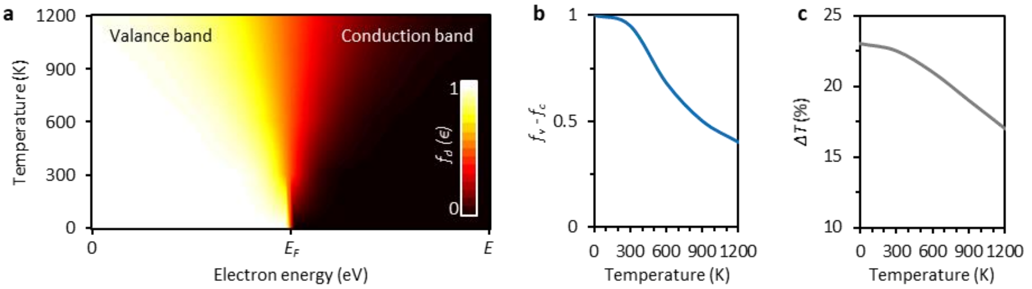

Figure S5 | Thermoelectric effect for Dirac Fermion . a, Calculation of the temperature tunable $f_d(\epsilon)$. **b,** the energy distribution possibility in valance band and in conductivity band varies with temperature. **c,** The temperature increase is potential to decrease the modulation depth in principle.

S2. Device fabrication and properties.

Figure S6 demonstrates the fabrication flow of our GFP resonator. Erbium/Ytterbium doped single mode fiber (EYDF, Nufern) was used to provide the gain, with 980 nm/1550 nm pump-gain efficiency > 30%. The insertion loss of the the EYDF and standard SMF-28e is under 0.12 dB. The all fiber section was carefully cut and capsuled in a ceramic adapter. Before further process, each fiber end of the cavity was polished to mirror smoothness, with spatial alignment inaccuracy < 1% arc-second (**Figure S6a**). The length of the fiber F-P resonator is fixed in 1 cm. The gain of the short Er^{3+}/Yb^{3+} based F-P cavity is much lower than a typical fiber loop based laser cavity, thus we had to ensure the high Q resonance.

Multilayer dielectric reflective film based distributed Bragg reflectors (DBRs) were composed on one side of the cavity (10-layer pairs of Ta_2O_5 and SiO_2 with a total thickness of 4.5 μm) first. Reflectivity of the DBR was higher than 99% (**Figure S6b**). Monolayer graphene on copper foils were grown by chemical vapor deposition method (CVD). A layer of PMMA protection film was coated by spin coating (3000r/min for 30 seconds) on the surface of the foil and baked at 150 °C (**Figure S6c**). Then, free-standing PMMA-supported graphene film is obtained by etching the bottom copper foil with $FeCl_3$ solution and wet transferred onto the surface of the fiber section, and the PMMA is further dissolved using acetone (**Figure S6d and S6e**). A customer designed soft stencil lithography make is then physically aligned and transferred on top of the erbium doped high Q factor fiber Fabry-Perot

resonator, then the Ti/Au contact electrodes (5 nm / 50 nm) of graphene were fabricated through e-beam evaporation (**Figure S6f**). Finally the other DBRs mirror coated on a fiber end was composed on the graphene, forming the F-P cavity (**Figure S6g**).

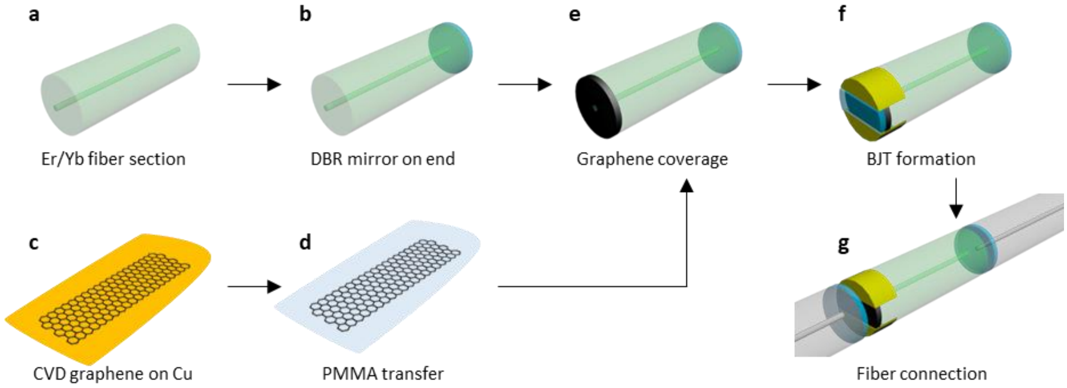

Figure S6 | Device fabrication. Graphene was grown via CVD technique and transferred on the micro fiber resonator, then formed the graphene heterostructure with electrical tunability.

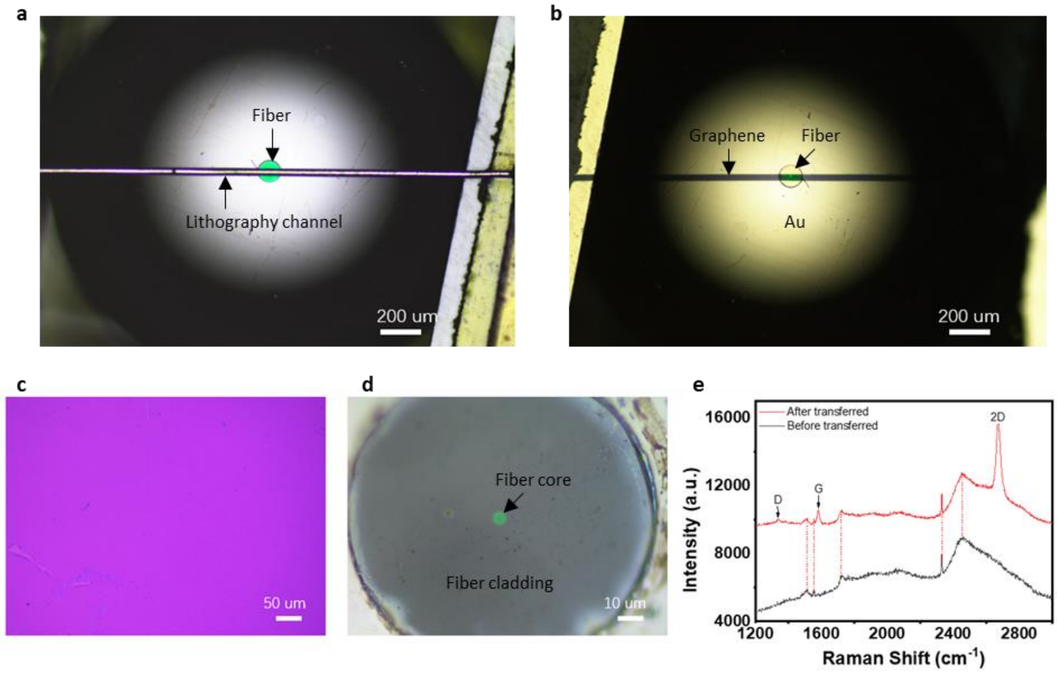

Figure S7 | Characterization of the graphene film on fiber. a & b, Optical image of the stencil lithography mask integrated on top of the graphene on fiber, and the graphene device after contact metal integration. Here the graphene, fiber and Au electrodes are marked. **c & d,** Microscopic picture of monolayer graphene on silicon wafer and on fiber. **e,** Raman spectroscopy of fiber surface before and after transferred monolayer graphene.

Figure S7 demonstrates the characterizations of the graphene on fiber. **Figure S7a** and **S7b** shows the pictures how we implemented the customer designed soft stencil lithography. The mask was carefully calibrated at the fiber core region, with proper width (≈ 60 μm). Such channel width enables acceptable tuning voltage as well as proper optical absorption. **Figure S7c** shows the optical microscopic photograph of the graphene, illustrating the uniformity. **Figure S7d** shows the optical image of the graphene, transferring kept the quality. **Figure S7e** demonstrates the Raman spectra of the fiber end, before and after graphene transferring. Very weak D peak, narrow G peak and 2D peak suggests the monolayer with few defects.

S3. Setups and extended measurements.

Figure S8a shows the setup to measure the optical transmission spectrum by using a low power laser (below the nonlinear excitation threshold). A tunable laser (Agilent 8168A) with stable CW output and typical linewidth 300 kHz scans the wavelength from 1520 nm to 1610 nm. In the transmission measurement, the output power of the tunable laser is fixed at 1 mW (below the ring-down threshold). The tunable CW laser beam is delivered into the GFPR directly, whose polarization can be fixed by a fiber polarization controller. Finally the transmission is detected by an IR band photodetector (PD, Thorlabs, 10 GHz) and then checked by a triggered oscilloscope with 500 MHz bandwidth (Tektronix TDS3052C). **Figure S8b** shows the measured spectrum of our dispersion engineered EDF F-P resonator, with FSR 10 GHz, and **Figure S8c** the Lorentz fitted single resonances, the blue and red curves show the cavity before and after the graphene incorporation. The graphene deposition induced optical absorption, total loss of the cavity changes from ≈ 6.2% to ≈ 8.5%, thus the intrinsic Q factor decreases from 1.9×10^6 to 1.4×10^6. This also verifies that the graphene induced loss is πα per layer. **In Figure S8c**, we also plot these resonances driven by current (grey curves). By increasing the graphene Fermi level, the intrinsic Q factor is tunable ranging from 1.3×10^6 to 1.6×10^6. **Figure S8d** plots the correlation of the round-trip transmissions and the resonance depth, obeying $T = (\alpha-|t|)^2/(1-\alpha|t|)^2$ [S42]. Here, 1−α is the cavity loss per round trip, and 1−t is the bus-to-cavity coupling rate. The SMF to cavity coupling efficiency is $t \approx 3\%$. In our experiment, the GFP resonator is tuned in the under-coupled region.

Figure S9a shows the setup to measure the saturable absorption. A high power femtosecond laser (mode locked fiber laser with 37 MHz repetition rate and 500 fs pulse delay) provides the high peak power used. Maximum peak power of the femtosecond laser is 2.2 kW, indicating the maximum 4.4 GW/cm^2 power density in the implementation, fulfilling the saturation process. The polarization controller (PC) is used to optimize and stabilize the polarization. A tunable attenuator is used to change the launched power. The GFPR behind the attenuator is controlled by the voltage tuning, finally the output power is detected by a high accuracy power meter. The current is controlled by the V_{SD}. In **Figure S9b**, the microscopic picture show the graphene channel lightened by the high power. The nonlinear reflection of the graphene based DBR would be checked by the power meter.

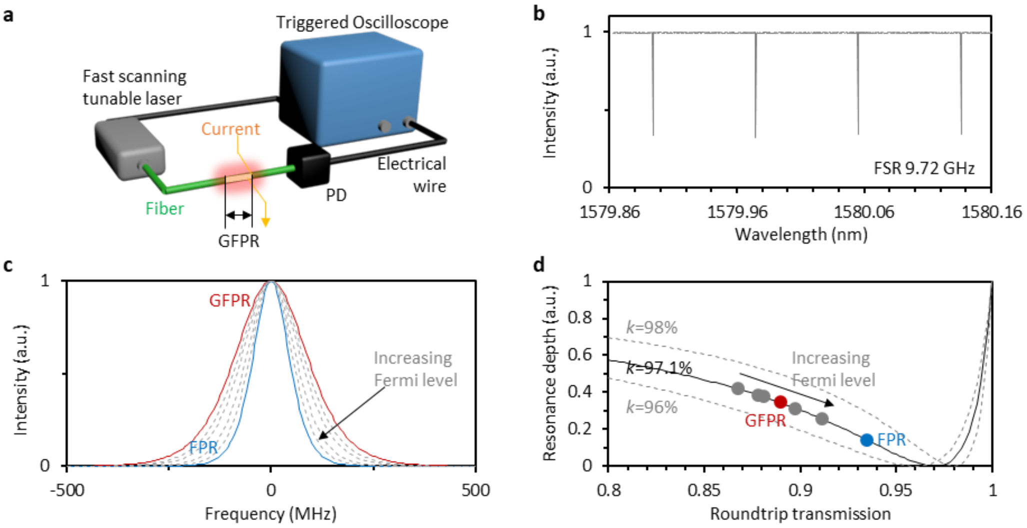

Figure S8 | Transmission measurements of the 10 GHz resonators. a, Fast resonance scan setup, PD: photodetector. **b,** Typical transmission FSR of a GFPR. **c,** Typical Lorentz fitted resonances for intrinsic Q, **d,** Transmission correlations.

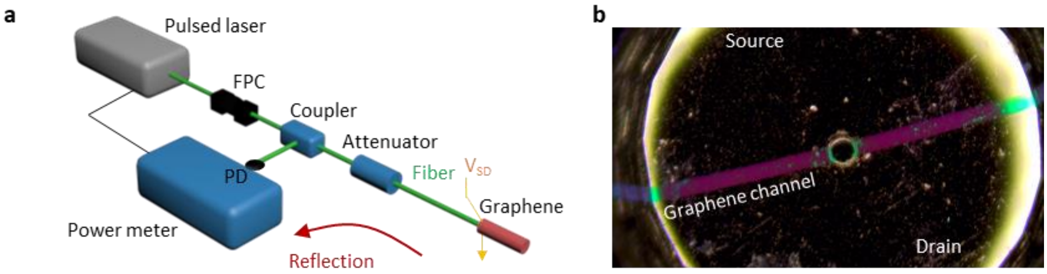

Figure S9 | Saturation absorption measurement. a, Setup for measuring the electrically controllable SA of graphene. In this figure, FPC: fiber polarization controller, PD: photodetector. **b,** Microscopic picture, here the graphene channel between the Au patterns is bright.

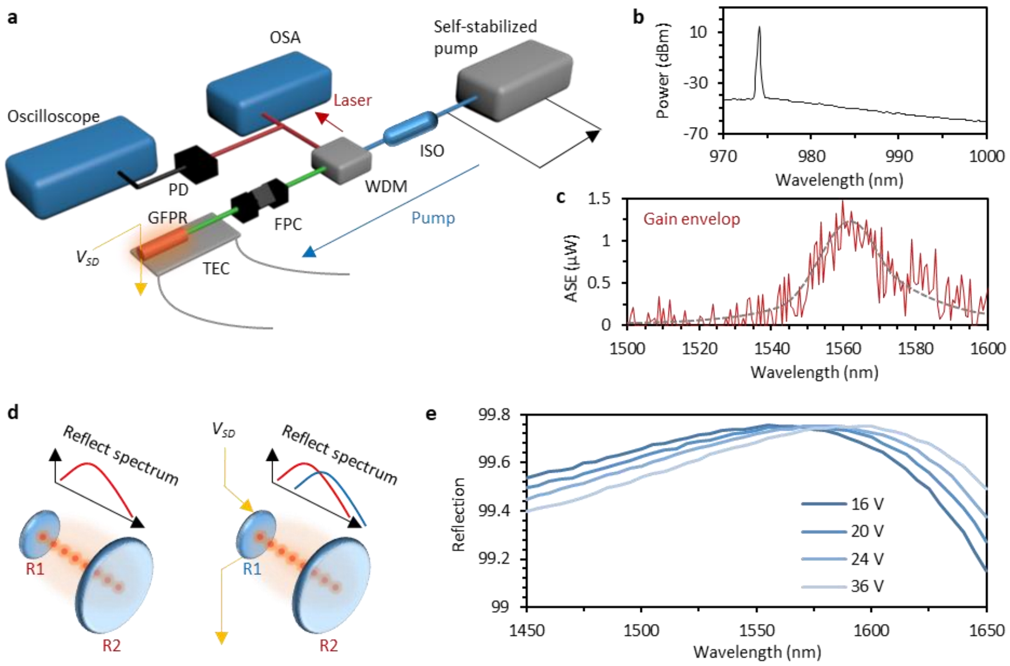

Figure S10 | Laser implementation. a, Setup for the laser driving. OSA: Optical spectrum analyzer, PD: photodetector, TEC: thermal energy converter, FPC: fiber polarization controller, WDM: wavelength division multiplexer, ISO: isolator, **b,** Typical spectrum of the pump. **c,** Measured ASE of the GFPR. **d,** Schematic diagram shows that tuning the graphene on one DBR side induces imbalanced reflection spectrally. **e,** The increment of V_{SD} enables central wavelength of the reflection red-shifting.

Figure S10a shows the setup for laser generation and control. A single mode and stable 980 nm laser diode is used as the optical pump, with maximum power 1.5 W. The pump is self-stabilized by the inner-integrated power feedback loop. An isolator protects the pump laser. Laser can be detected either in transmission or reflection direction, by using a 980/1550 wave division multiplexer (WDM). Bandwidth of the WDM covers from 1520 nm to 1610 nm. Then a fiber polarization controller fixes the input polarization linearly, to avoid the instability caused by the birefringence. The GFPR is fixed on a V-groove fixture with a thermal energy converter (TEC). The temperature controlling resolution of the TEC is determined by the controller (Thorlabs TC 200, 10 mK). V_{SD} applies on the graphene covered side of the resonator. Dynamics of the laser could be monitored by both an optical spectrum analyzer (OSA, Yokogawa 6370D), and an oscilloscope after the PD. **Figure S10b** shows the spectrum of the 980 nm pump, which is clean spectrally, with central wavelength 974.1 nm and SNR over 50

dB. **Figure S10c** shows the ASE of the doped fiber, which provides wide gain from 1550 nm to 1580 nm. **Figure S10d** shows the schematic diagram that changing the V_{SD} leads to the unbalance reflection of the two DBR facets. **Figure S10e** plots the measured reflection spectral envelope of our GFPR. The increment of V_{SD} red-shifts its wavelength of reflection peak. Here we normalize the graphene induced absorption.

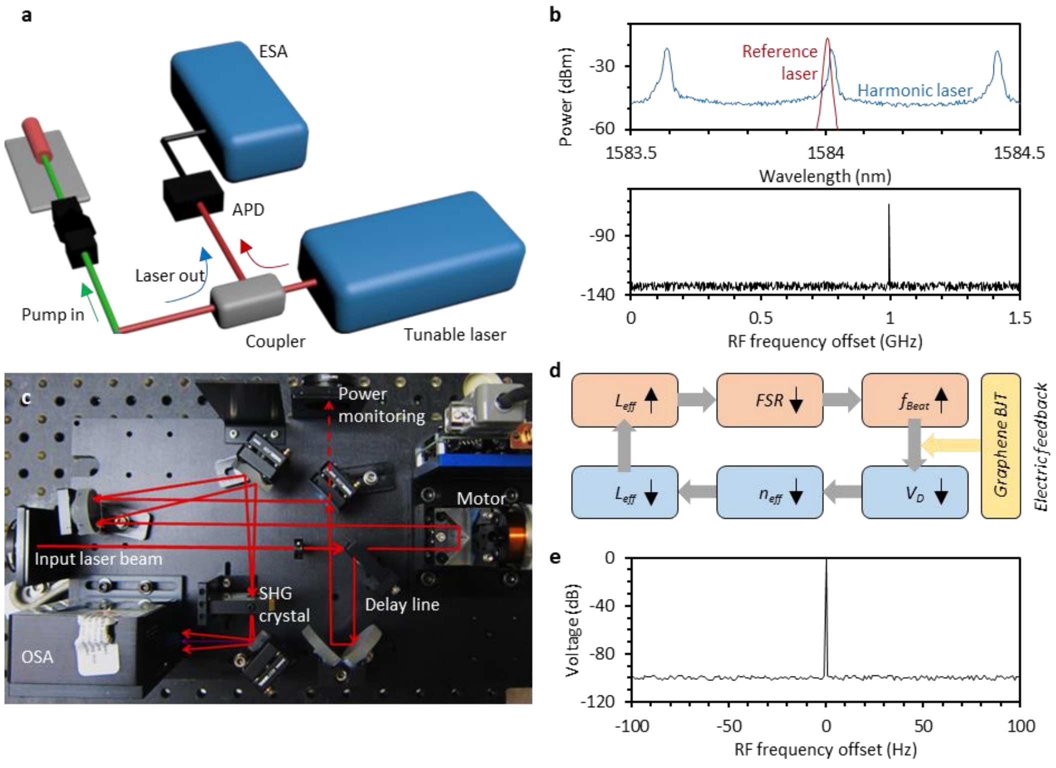

Figure S11 | Ultrafast laser characterization and stabilization. a, Setup for heterodyne beating. ESA: electric spectrum analyzer. **b,** Setup for frequency resolved autocorrelation measurement. SHG: second harmonic generation. **c,** Scheme of graphene based optoelectronic negative-feedback stabilization for the ultrafast laser. **d,** Typical spectrum of the RF reference signal.

Figure S11a shows the setup for measuring the beat notes. A commercial tunable laser with CW operation (Agilent 8168A, 200 kHz linewidth) serves as the reference. The difference frequency of the laser line and the reference line Δf could be well shifted to hundred MHz level, which is convenient for amplified by using a 10 GHz PD. An electric spectrum analyzer (Rohde & Schwarz, 2 ~ 43.5 GHz) provides the high-resolution RF measurement. **Figure S11b** shows the implementation. We tune the

reference laser approaching to one comb line first, this generates a beat note of the comb line and the reference laser, at sub GHz frequency. Then we check the noise of the beat note. **Figure S11c** shows our setup for the frequency-resolved autocorrelation measurement, taking advantage of a BBO crystal to generate SHG. Spectral resolution of the OSA here is 0.2 nm. The maximum delay is 32 ps. Moreover, in the experiment, we also use the graphene heterostructure for further stabilizing the resonator, to achieve better locking. **Figure S11d** sketches principle of the graphene based negative feedback scheme, The FSR uncertainty in the resonator majorly comes from the instability of the effective cavity length L_{eff}, which is determined by the mode effective index of the intra-cavity media n_{eff} and the thermal expansion. These two aspects interact with the intra-cavity optical power. By stabilizing the intra cavity power via controlling the absorption of the graphene dynamically, we can stabilize the f_{FSR} finally. Here a stable RF synthesizer (Anritsu MG3694B) is used as the reference. **Figure S11e** shows the generated RF signal from the synthsizer, with linewidth in single Hz level.

In **Figure S12a**, we show the scheme to amplify and span the mode locked laser, then stabilize the *FSR* by using RF heterodyne beating. The EDFAs have maximum amplification rate 40 dB, it can amplify the mode locked pulse energy from 60 fJ to 50 pJ. Then the amplified combs is spanned by an 8 m long dispersion shifted highly nonlinear fiber (DS-HNLF, core diameter 4 μm) with a nonlinear coefficient of 0.04 /Wm and zero dispersion around 1570nm. The EDFAs induces both white frequency noise and technical noise. The stable RF synthesizer (Anritsu MG3694B) is used for locking the f_{FSR} of the laser, the beat note of the laser and the RF synthesizer is sent to a loop filter and then an electric amplifier. This process is similar to a lock-in amplification. Afterwards the amplified voltage is added on the GPFR negatively, for compensating the V_{SD}. We note that this feedback loop is both sensitive to the beat note shift and the amplitude alteration, thus intense intensity jitter should be suppressed by using TEC first. **Figure S12b** plots the generated laser comb before supercontinuum broadening in log-scale. Before, its envelope is in sech^2 shape, with bandwidth is 3.6 nm (≈ 4.5 THz). **Figure S12c** plots the SSB phase noise of the generated laser comb before super-continuum broadening. The free running laser comb shows a SSB phase noise -80 dBc/Hz@1 kHz, and -110 dBc/Hz@10 kHz. By implementing the graphene based stabilization, we promote the performance to -95 dBc/Hz@1 kHz, and -127 dBc/Hz@10 kHz. The phase noise in the compact device is 7 dBc/Hz better than the super-

continuum broadened comb, as the amplifying and broadening brings external noise in.

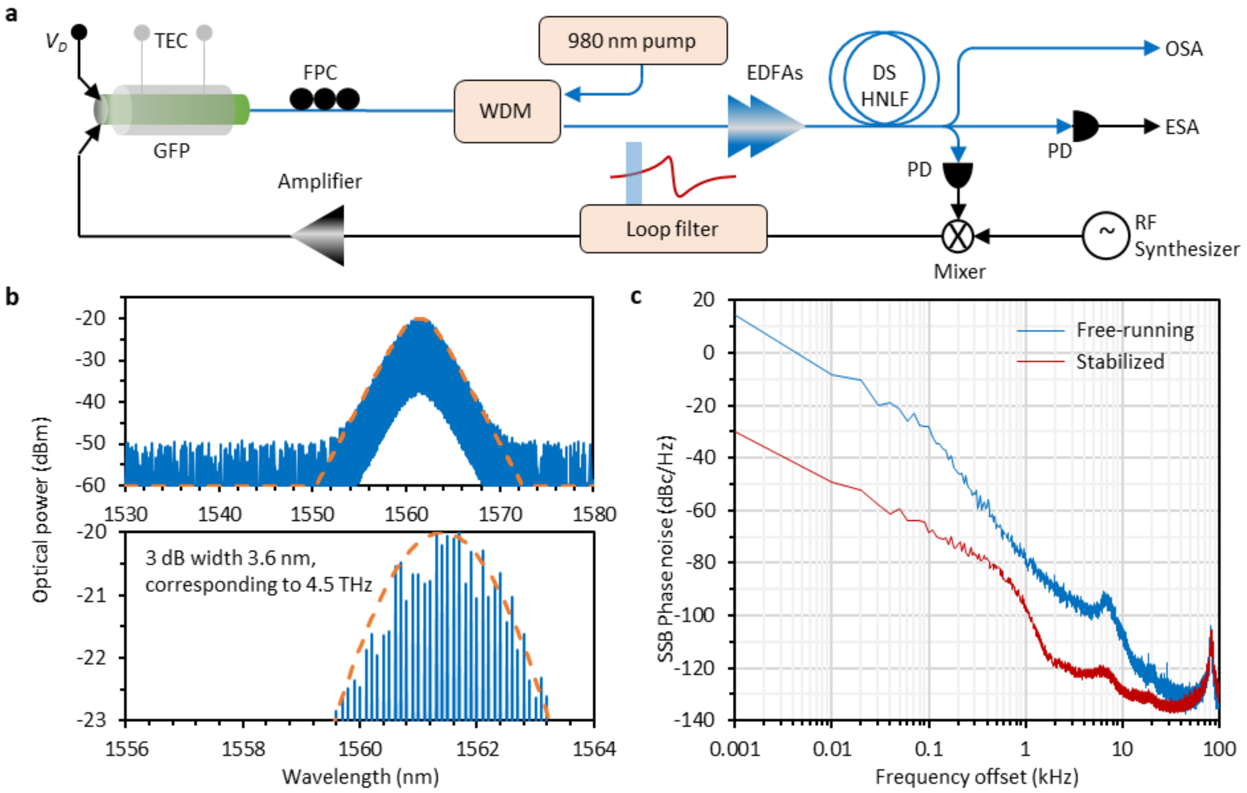

Figure S12 | Setup of comb spanning and feedback stabilization. a, By using the RF synthesizer based frequency down-conversion feedback loop linking to the GFPR directly, both the laser power and the comb frequency spacing can be stabilized. **b,** Spectrum of the mode locked fiber laser comb in log scale, here the orange dashed curve shows $sech^2$ fitting. **c,** Phase noise spectra of the laser comb before broadening. Locked on the RF synthesizer, *S(f)* is suppressed down to -127 dBc/Hz@10 kHz.

Table S5 demonstrates more results about the Q distribution in spectrum, corresponding to the Figure 2 in the maintext. When V_{SD} = 0 V, the highest Q appears at 1529.717 nm, around 1.43×10^6; while the lowest Q appears at 1559.336 nm, around 1.39×10^6. When V_{SD} = 10 V, the highest loaded Q appears at 1526.704 nm, around Q 1.46×10^6; while the lowest Q appears at 1577.478 nm, around Q 1.42×10^6. When V_{SD} = 20 V, the highest Q appears at 1568.542 nm, around Q 1.49×10^6; while the lowest Q appears at 1525.103 nm, around Q 1.45×10^6. When V_{SD} = 30 V, the highest Q appears at 1578.663 nm, around Q 1.56×10^6; while the lowest Q appears at 1524.943 nm, around Q 1.52×10^6. When V_{SD} = 40 V, the highest Q appears at 1582.819 nm, around Q 1.61×10^6; while the lowest Q

appears at 1528.228 nm, around Q 1.57×10^6.

Table S5 | Intrinsic Q distribution (Unit: million)

**λ (nm±5nm)**	**1520**	**1530**	**1540**	**1550**	**1560**	**1570**	**1580**	**1590**	**1600**
**V_{SD} 0 V**	1.42	**1.43**	1.42	1.40	1.39	1.41	1.41	1.4	1.4
**V_{SD} 10 V**	1.45	**1.46**	1.43	1.44	1.45	1.44	1.42	1.43	1.43
**V_{SD} 20 V**	1.45	1.45	1.46	1.45	1.47	**1.49**	1.48	1.47	1.46
**V_{SD} 30 V**	1.50	1.52	1.52	1.53	1.55	1.54	**1.56**	1.54	1.55
**V_{SD} 40 V**	1.55	1.57	1.57	1.58	1.58	1.6	1.6	**1.61**	1.59
**V_{SD} 50 V**	1.58	1.58	1.59	1.60	1.60	1.60	1.61	1.61	**1.62**

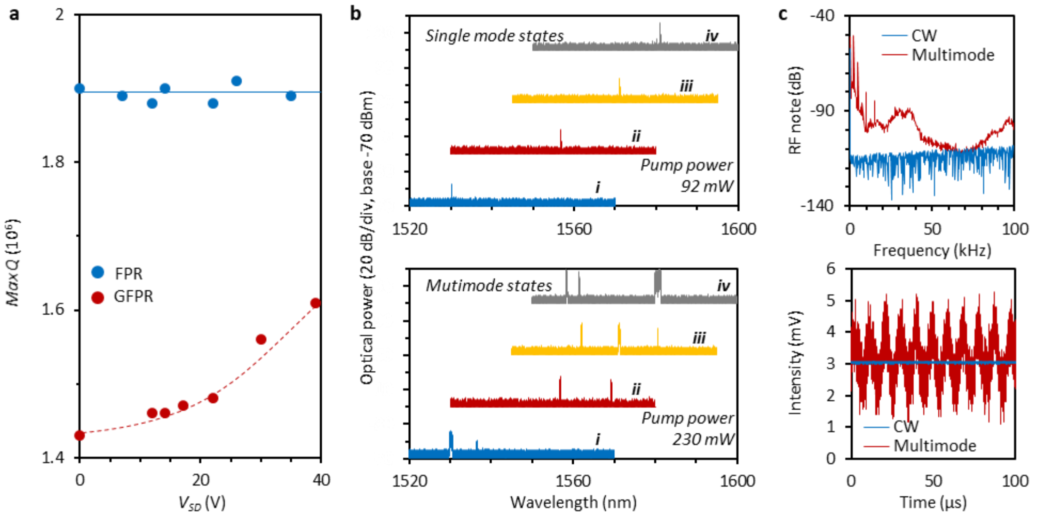

Figure S13 | Electrical tuning comparisons. a, Determined by the tunable absorption, the GFPR is electrically tunable, while the FPR without graphene is not. **b,** Electrical tuning of wavelength for CW operation and multimode operation. **c,** Time-frequency characterization of a typical CW laser generation and multimode laser generation.

Figure S13a compares the electric tunability on the maximum Q, for the GFPR and the FPR without graphene integration respectively. For a silica fiber section without graphene, there is no surface current, thus there is no transmission tunability. **Figure S13b** compares the spectra of the single mode states under 92 mW pump power (top panel), and the multiple mode states under 230 mW pump power (bottom panel). The multiple longitude modes generation is the premise of mode locking. Before

locking state, the longitude modes are not stable. **Figure S13c** iprovide the typical low-frequency amplitude noise spectra of a single mode state and a multimode state in radio frequency (RF) in the range of 0 to 100 kHz, and their temporal traces correspondingly. For a single mode output, the amplitude noise is considerably low (-120 dBm), limited by the pump stability and the SNR of the photodetector, it outputs the continuous wave in time domain. On the other hand, for a multimode state below the locking power, the inter-mode competition is fierce, each FSR may suffer gain instability. As a result, the RF demonstrates high noise, so does the temporal trace. To suppress the noise down while keeping the multiple laser lines stable like a frequency comb, saturable absorption and cavity stabilization for soliton mode locking would make sense.

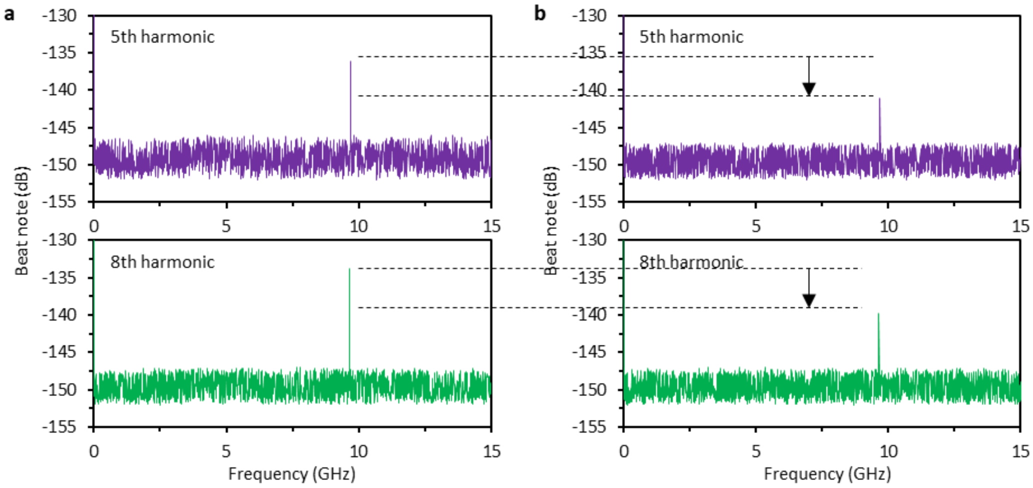

Figure S14 | Suppressing the super-mode noise. a, Laser beat notes at ≈ 10 GHz show there exists super-mode noises in the harmonic locking states. **b,** By decreasing the V_{SD} carefully, the super-mode noise could be suppressed, due to the better longitude mode selectivity.

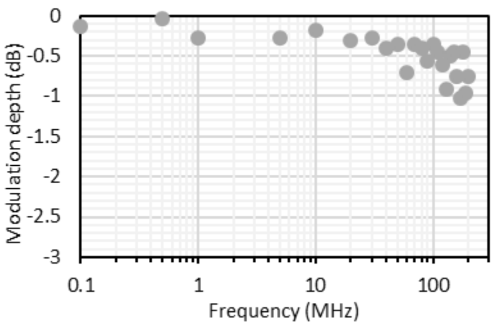

Figure S15 | Modulation bandwidth of the graphene heterostructure. Limited by the signal generator, the optical modulation depth keeps > -1 dB (≈ 80%) in the range of 0 to 200 MHz.

Super-mode noise in the harmonic mode locking states cannot be properly shown in heterodyne

measurement in the MHz range. By using a high-speed photodetector (15 GHz), we characterize the beat note of the fifth order harmonic locking state and the eighth order harmonic locking state, in **Figure S14a**. Here the first-order super-mode beating line, located at 10 GHz, is observable. When free running, a higher harmonic order can induce higher super-mode noise. For suppressing the super-mode noise in this GFPR, a simple method is to increase the saturation threshold of the graphene. This can optimize the longitude mode selectivity. Referring the results shown in Fig. 2 in maintext, such a saturation threshold increment could be realized just by decreasing the V_{SD} a bit carefully. Keeping the harmonic locking state and changing the V_{SD}, we can suppress the 1st order super-mode noise beat note over 6 dB (**Figure S14b**). To test the modulation bandwidth of the graphene heterostructure, we use a tunable signal generator controlling its optical transparency (sinusoidal waveform, *Vpp* 20 V to 40 V, light wavelength 1560 nm, power 1 mW, far below the saturation threshold). **Figure S15** plots the spectral response. The measured bandwidth is over 200 MHz, just limited by the signal generator. Here the modulation depth is normalized by the DC signal.

Supplementary references

1. Martinez, A. & Yamashita, S. 10 GHz fundamental mode fiber laser using a graphene saturable absorber. *Appl. Phys. Lett.* **101**, 041118 (2012).
2. He, W., Pang, M., Menyuk, C. R. & St. J. Russell, P. Sub-100-fs 187 GHz mode-locked fiber laser using stretched-soliton effects. *Optica* **3**, 1366 (2016).
3. Sotor, J. *et al.* All-fiber Ho-doped mode-locked oscillator based on a graphene saturable absorber. *Opt. Lett.* **41**, 2592 (2016).
4. Mary, R. *et al.* 15 GHz picosecond pulse generation from a monolithic waveguide laser with a graphene-film saturable output coupler. *Opt. Express* **21**, 7943 (2013).
5. Lagatsky, A. A. *et al.* Fundamentally mode-locked, femtosecond waveguide oscillators with multi-gigahertz repetition frequencies up to 15 GHz. *Opt. Express* **21**, 19608 (2013).
6. Liu, S. *et al.* Characterization of a fully integrated heterogeneous silicon/III-V colliding pulse mode-locked laser with on-chip feedback. *Opt. Express* **26**, 9714 (2018).
7. Hou, L., Haji, M., Akbar, J., Qiu, B. & Bryce, A. C. Low divergence angle and low jitter 40 GHz AlGaInAs/InP 155 μm mode-locked lasers. *Opt. Lett.* **36**, 966 (2011).

8. Lee, E. J. *et al.* Active control of all-fibre graphene devices with electrical gating. *Nat. Commun.* **6**, 6851 (2015).

9. Li, C. *et al.* Manipulation of nonlinear optical properties of graphene bonded fiber devices by thermally engineering fermi–dirac distribution. *Adv. Opt. Mater.* **5**, 1–6 (2017).

10. Bogusławski, J. *et al.* Graphene actively mode-locked lasers. *Adv. Funct. Mater.* **28**, 1–7 (2018).

11. Xue, X., Zheng, X. & Zhou, B. Super-efficient temporal solitons in mutually coupled optical cavities. *Nat. Photonics* **13**, 616–622 (2019).

12. Huang, S.-W. *et al.* A broadband chip-scale optical frequency synthesizer at 2.7 x 10-16 relative uncertainty. *Sci. Adv.* **2**, e1501489–e1501489 (2016).

13. Del'Haye, P. *et al.* Phase-coherent microwave-to-optical link with a self-referenced microcomb. *Nat. Photonics* **10**, 516–520 (2016).

14. Liang, W. *et al.* High spectral purity Kerr frequency comb radio frequency photonic oscillator. *Nat. Commun.* **6**, 7957 (2015).

15. Pavlov, N. G. *et al.* Narrow-linewidth lasing and soliton Kerr microcombs with ordinary laser diodes. *Nat. Photonics* **12**, 694–698 (2018).

16. Lucas, E. *et al.* Ultralow-noise photonic microwave synthesis using a soliton microcomb-based transfer oscillator. *Nat. Commun.* **11**, 374 (2020).

17. Zhang, M. *et al.* Broadband electro-optic frequency comb generation in a lithium niobate microring resonator. *Nature* **568**, 373–377 (2019).

18. Rueda, A., Sedlmeir, F., Kumari, M., Leuchs, G. & Schwefel, H. G. L. Resonant electro-optic frequency comb. *Nature* **568**, 378–381 (2019).

19. Mikhailov, S. A. & Ziegler, K. New electromagnetic mode in graphene. *Phys. Rev. Lett.* **99**, 016803 (2007).

20. Marini, A., Cox, J. D. & Garc á De Abajo, F. J. Theory of graphene saturable absorption. *Phys. Rev. B* **95**, 1–11 (2017).

21. Sun, Z. *et al.* Graphene mode-locked ultrafast laser. *ACS Nano* **4**, 803–810 (2010).

22. Kampfrath, T., Perfetti, L., Schapper, F., Frischkorn, C. & Wolf, M. Strongly Coupled Optical

Phonons in the Ultrafast Dynamics of the Electronic Energy and Current Relaxation in Graphite. *Phys. Rev. Lett.* **95**, 187403 (2005).

23. Balandin, A. A. Thermal properties of graphene and nanostructured carbon materials. *Nat. Mater.* **10**, 569–581 (2011).
24. Brida, D. *et al.* Ultrafast collinear scattering and carrier multiplication in graphene. *Nat. Commun.* **4**, 1–9 (2013).
25. Soavi, G. *et al.* Broadband, electrically tunable third-harmonic generation in graphene. *Nat. Nanotechnol.* **13**, 583–588 (2018).
26. Schwierz, F. Graphene transistors. *Nat. Nanotechnol.* **5**, 487–496 (2010).
27. Das, A. *et al.* Monitoring dopants by Raman scattering in an electrochemically top-gated graphene transistor. *Nat. Nanotechnol.* **3**, 210–215 (2008).
28. Bao, Q. *et al.* Atomic-layer graphene as a saturable absorber for ultrafast pulsed lasers. *Adv. Funct. Mater.* **19**, 3077–3083 (2009).
29. Wu, Y. *et al.* Graphene-coated microfiber Bragg grating for high-sensitivity gas sensing. *Opt. Lett.* **39**, 1235 (2014).
30. Ran, Z. L., Rao, Y. J., Liu, W. J., Liao, X. & Chiang, K. S. Laser-micromachined Fabry-Perot optical fiber tip sensor for high-resolution temperature-independent measurement of refractive index. *Opt. Express* **16**, 2252 (2008).
31. Quiroga-Teixeiro, M., Clausen, C. B., Sørensen, M. P., Christiansen, P. L. & Andrekson, P. A. Passive mode locking by dissipative four-wave mixing. *J. Opt. Soc. Am. B* **15**, 1315 (1998).
32. Hönninger, C., Paschotta, R., Morier-Genoud, F., Moser, M. & Keller, U. Q-switching stability limits of continuous-wave passive mode locking. *J. Opt. Soc. Am. B* **16**, 46 (1999).
33. Grudinin, A. B. & Gray, S. Passive harmonic mode locking in soliton fiber lasers. *J. Opt. Soc. Am. B* **14**, 144 (1997).
34. Agrawal, G. *Nonlinear Fiber Optics*. *Nonlinear Fiber Optics* (2012). doi:10.1016/C2011-0-00045-5
35. Koke, S. *et al.* Direct frequency comb synthesis with arbitrary offset and shot-noise-limited phase noise. *Nat. Photonics* **4**, 462–465 (2010).

36. Kärtner, F. X., Jung, I. D. & Keller, U. Soliton mode-locking with saturable absorbers. *IEEE J. Sel. Top. Quantum Electron.* **2**, 540–556 (1996).

37. Kelly, S. M. J. Characteristic sideband instability of periodically amplified average soliton. *Electron. Lett.* **28**, 806 (1992).

38. Dennis, M. L. & Duling, I. N. Experimental Study of Sideband Generation in Femtosecond Fiber Lasers. *IEEE J. Quantum Electron.* **30**, 1469–1477 (1994).

39. Law, S. Y. & Agrawal, G. P. Effects of spatial hole burning on gain switching in vertical-cavity surface-emitting lasers. *IEEE J. Quantum Electron.* **33**, 462–468 (1997).

40. Huang, S.-W. *et al.* A broadband chip-scale optical frequency synthesizer at $2.7 \times 10 -16$ relative uncertainty. *Sci. Adv.* **2**, e1501489 (2016).

41. Freitag, M., Low, T., Xia, F. & Avouris, P. Photoconductivity of biased graphene. *Nat. Photonics* **7**, 53–59 (2013).

42. Yariv, A. Critical coupling and its control in optical waveguide-ring resonator systems. *IEEE Photonics Technol. Lett.* **14**, 483–485 (2002).
